# Cost-utility analysis of four WHO-recommended sofosbuvir-based regimens for the treatment of chronic hepatitis C in sub-Saharan Africa

**DOI:** 10.1186/s12913-021-07289-0

**Published:** 2022-03-05

**Authors:** Sylvie Boyer, Maël Baudoin, Marie Libérée Nishimwe, Melina Santos, Maud Lemoine, Gwenaëlle Maradan, Babacar Sylla, Charles Kouanfack, Patrizia Carrieri, Abbas Mourad, Nicolas Rouveau, Raoul Moh, Moussa Seydi, Alain Attia, Maame Esi Woode, Karine Lacombe

**Affiliations:** 1 Aix Marseille Univ, Inserm, IRD, SESSTIM, Sciences Economiques & Sociales de la Santé & Traitement de l’Information Médicale, ISSPAM, Faculté de Médecine, 27 Bd Jean Moulin, 13 005 Marseille, France; 2 ORS PACA, Observatoire régional de la santé Provence-Alpes-Côte d’Azur, Marseille, France; 3 Department of Metabolism, Digestion and Reproduction, Division of Digestive Diseases, Section of Hepatology and Gastroenterology, St Mary’s Hospital, Imperial College, London, UK; 4 IMEA, Paris, France; 5 Hôpital de Jour, Hôpital Central de Yaoundé, Yaoundé, Cameroon; 6 Faculté de Médecine et des Sciences Pharmaceutiques, Université de Dschang, Dschang, Cameroon; 7 ANRS, Paris, France; 8 MEREVA, PACCI Abidjan, Abidjan, Côte d’Ivoire; 9 Unité Pédagogique de Dermatologie et Infectiologie, UFR des sciences médicales, Abidjan, Côte d’Ivoire; 10 Service des Maladies Infectieuses et Tropicales, CHU Fann, Dakar, Sénégal; 11 Service d’hépatologie, CHU Yopougon, Abidjan, Côte d’Ivoire; 12 Centre for Health Economics, Monash University, Melbourne, Australia; 13 Sorbonne Université, INSERM, Institut Pierre Louis d’Epidémiologie et de Santé Publique, IPLESP, Paris, France; 14 APHP, Hôpital Saint-Antoine, Service de Maladies Infectieuses et Tropicales, Paris, France

**Keywords:** Chronic hepatitis C, Sofosbuvir, Direct-acting antivirals, Cost-effectiveness analysis, Cameroon, Senegal, Côte d’Ivoire, Cost-utility analysis

## Abstract

**Background:**

Although direct-acting antivirals (DAA) have become standard care for patients with chronic hepatitis C worldwide, there is no evidence for their value for money in sub-Saharan Africa. We assessed the cost-effectiveness of four sofosbuvir-based regimens recommended by the World Health Organization (WHO) in Cameroon, Côte d’Ivoire and Senegal.

**Methods:**

Using modelling, we simulated chronic hepatitis C progression with and without treatment in hypothetical cohorts of patients infected with the country’s predominant genotypes (1, 2 and 4) and without other viral coinfections, history of liver complication or hepatocellular carcinoma. Using the status-quo ‘no DAA treatment’ as a comparator, we assessed four regimens: sofosbuvir-ribavirin, sofosbuvir-ledipasvir (both recommended in WHO 2016 guidelines and assessed in the TAC pilot trial conducted in Cameroon, Côte d’Ivoire and Senegal), sofosbuvir-daclatasvir and sofosbuvir-ledipasvir (two pangenotypic regimens recommended in WHO 2018 guidelines). DAA effectiveness, costs and utilities were mainly estimated using data from the TAC pilot trial. Secondary data from the literature was used to estimate disease progression probabilities with and without treatment. We considered two DAA pricing scenarios: S1) originator prices; S2) generic prices. Uncertainty was addressed using probabilistic and deterministic sensitivity analyses and cost-effectiveness acceptability curves.

**Results:**

With slightly higher effectiveness and significantly lower costs, sofosbuvir/velpatasvir was the preferred DAA regimen in S1 with incremental cost-effectiveness ratios (ICERs) ranging from US$526 to US$632/QALY. At the cost-effectiveness threshold (CET) of 0.5 times the 2017 country’s per-capita gross domestic product (GDP), sofosbuvir/velpatasvir was only cost-effective in Senegal (probability > 95%). In S2 at generic prices, sofosbuvir/daclatasvir was the preferred regimen due to significantly lower costs. ICERs ranged from US$139 to US$216/QALY according to country i.e. a 95% probability of being cost-effective. Furthermore, this regimen was cost-effective (probability> 95%) for all CET higher than US$281/QALY, US$223/QALY and US$195/QALY in Cameroon, Côte d’Ivoire and Senegal, respectively, corresponding to 0.14 (Côte d’Ivoire and Senegal) and 0.2 (Cameroon) times the country’s per-capita GDP.

**Conclusions:**

Generic sofosbuvir/daclatasvir is very cost-effective for treating chronic hepatitis C in sub-Saharan Africa. Large-scale use of generics and an increase in national and international funding for hepatitis C treatment must be priorities for the HCV elimination agenda.

**Supplementary Information:**

The online version contains supplementary material available at 10.1186/s12913-021-07289-0.

## Background

Chronic hepatitis C (CHC) is a lifelong infection caused by the hepatitis C virus (HCV) which may progress to liver fibrosis, cirrhosis and hepatocellular carcinoma (HCC) without timely diagnosis and adequate treatment [1]. Of the estimated 71 million people with CHC worldwide [2], 10 million (14%) live in sub-Saharan Africa (SSA), including 7.5 million in West and Central Africa [3]. Despite being one of the regions most affected by HCV, SSA has the lowest treatment coverage in the world. The World Health Organization (WHO) estimates that approximately 4% of people with CHC there have been diagnosed and only 2% of these treated, giving a treatment coverage near zero [2]. Consequently, HCV-related morbidity and mortality are particularly high in SSA [4]: in 2015-2016, HCV was responsible for 1,289,252 years of life lost in Africa [5] and specifically 39,000 HCC-related deaths in West and Central Africa [6].

The advent of second generation direct-acting antivirals (DAA) in 2014 radically changed the hepatitis C landscape. Trials in high-income countries showed sustained virologic response (SVR) rates > 90% [7–11]. Recently, short-term efficacy and tolerability were also successfully reported in West, Central [12], and East [13] Africa. Furthermore, their favourable toxicity profile and short treatment duration (8-16 weeks) makes them easier to manage than interferon-based regimens, both for healthcare professionals and patients.

Today, DAA have replaced interferon-based regimens as standard care for CHC patients worldwide and are considered an indispensable tool to reach the WHO’s targets of HCV elimination by 2030 (namely a 80% treatment coverage in those eligible for treatment, a 90% reduction in incidence of new infections and a 65% reduction in liver-related mortality) [14].

The WHO 2016 guidelines recommending sofosbuvir/daclatasvir, sofosbuvir/ledipasvir and sofosbuvir/ribavirin for adults [15] were updated in 2018 with pangenotypic DAA regimens (sofosbuvir/daclatasvir, sofosbuvir/velpatasvir and glecaprevir/pibrentasvir) achieving high SVR rates (> 85%) across all seven major HCV genotypes [16].

Until recently, integrating DAA into national health systems in high-income countries was a controversial issue, because of their very high prices (e.g., in 2016, 12 weeks of treatment cost US$84,000 and US$76,720 per person in the USA and France, respectively). Despite this drawback, many economic evaluation studies - mainly in the USA and European countries - have demonstrated their economic value in this setting [17–19]. However, evidence is still lacking in low-income countries, especially in SSA where human, technical and financial resources are particularly restricted. Recently, agreements with the two DAA drug license owners (Gilead for sofosbuvir, ledipasvir and velpatasvir, and Bristol-Myers Squibb for daclatasvir) [20, 21] authorized the manufacture and sale of generic DAA at much lower costs in one hundred low-income countries. This is a major opportunity to better tackle the HCV disease burden by scaling-up access to DAA. Accordingly, identifying the optimal regimens to use in SSA in terms of economic value and affordability is essential to inform public health policy decisions.

Using a modelling approach combining data from the Treatment Africa Hepatitis C (TAC) pilot trial (ANRS 12311) and from the literature, we assessed the costs and cost-effectiveness of the four main sofosbuvir-based interferon-free DAA regimens recommended in the WHO 2016 and 2018 guidelines versus the status-quo, in Cameroon, Côte d’Ivoire and Senegal. We also discussed the affordability of scaling-up these regimens in regard to current government health expenditures in all three countries.

## Methods

A Markov cohort model was developed to simulate outcomes of CHC patients receiving DAA treatment or not in Cameroon, Côte d’Ivoire and Senegal. We used a lifetime horizon and discounted health outcomes and costs at an annual rate of 4% [22].

### Target population

In each study country, we considered a hypothetical cohort of 10,000 CHC patients infected with the country’s predominant genotypes (1, 2 and 4) [3, 12]. Patients were 55 years old and had no other viral coinfections, history of liver complication or HCC. The characteristics of the cohort at model entry are described in Table 1.

**Table 1:** Model parameters

Model parameters	Base-case value	Distribution	95% CI	Source
**Cohort characteristics (at model entry)**				
Age (years)	55	_	_	TAC trial [12]
Proportion of women	45.8	–	–	TAC trial [12]
Fibrosis initial stages^c^				
*F0*	0.076	_		[24]
*F1*	0.392			
*F2*	0.262			
*F3*	0.186			
*CC*	0.084			
**Effectiveness of the four studied sofosbuvir-based regimens**				
SVR12 SOF/RBV		–		TAC trial [12]
*Non Cirrhotic*	0.922		[0.820;0.999] ^b^	
*Cirrhotic*	0.793		[0.705;0.859] ^b^	
SVR12 SOF/LDV		–		TAC trial [12]
*Non Cirrhotic*	0.909		[0.832;0.973] ^b^	
*Cirrhotic*	0.782		[0.716;0.837] ^b^	
SVR12 SOF/DCV		–		HEPATHER cohort [28]
*Non Cirrhotic*	0.958		[0.895;1.000] ^b^	
*Cirrhotic*	0.824		[0.770;0.870] ^b^	
SVR12 SOF/VEL		–		HEPATHER cohort [28]
*Non Cirrhotic*	0.977		[0.913;1.000] ^b^	
*Cirrhotic*	0.840		[0.785;0.887] ^b^	
**Natural history of CHC: annual disease transition probabilities between health states in untreated, uncured or re-infected patients**				
All states → non-CHC related mortality^d^	_	_	_	[29]
F0 → F1	0.079	Beta(21.1;234.7)	[0.052;0.119]	[30]
F1 → F2	0.059	Beta(88.4;1399.1)	[0.048;0.072]	[30]
F2 → F3	0.108	Beta(30.0;238.7)	[0.077;0.152]	[30]
F3 → CC	0.077	Beta(14.8;164.1)	[0.047;0.127]	[30]
F3 → DC	0.012	Beta(7.0;558.1)	[0.005;0.023] ^a^	[31]
F3 → HCC	0.011	Beta(7.0;558.1)	[0.005;0.023] ^a^	[31]
F3 → CHC-related mortality	0.008	Beta(4.9;527.5)	[0.003;0.019] ^a^	[31]
CC → DC	0.041	Beta(99.5;2290.4)	[0.034;0.050] ^a^	[1]
CC → HCC	0.042	Beta(90.0;2048.7)	[0.034;0.051] ^a^	[1]
CC → CHC-related mortality	0.026	Beta(11.5;407.0)	[0.014;0.045] ^a^	[31]
DC → HCC	0.068	Beta(37.9;514.7)	[0.049;0.091] ^a^	[32]
DC → CHC-related mortality	0.130	Beta(75.7;489.9)	[0.107;0.163] ^a^	[32]
HCC → CHC-related mortality	0.900	Beta(186.3;19.9)	[0.86;0.94] ^a^	[33]
**CHC progression after DAA treatment in cured patients: annual disease transition probabilities between health states**				
CC → DC	0.023	Beta(14.1;540.5)	[0.014;0.040]^a^	[1]
CC → HCC	0.014	Beta(37.9;514.7)	[0.007;0.029]	[1]
CC → CHC-related mortality	0.026	Beta(11.5;407.0)	[0.014;0.045] ^a^	[31]
DC → HCC	0.068	Beta(37.9;514.7)	[0.049;0.091] ^a^	[32]
DC → CHC-related mortality	0.130	Beta(75.7;489.9)	[0.107;0.163] ^a^	[32]
**CHC progression after DAA treatment in cured patients: annual disease transition probabilities between health states**				
HCC → CHC-related mortality	0.900	Beta(186.3;19.9)	[0.86;0.94]	[33]
Annual reinfection probabilities				
*mono-infected*	0.002	Beta(13.2;7051.8)	[0.001;0.003]	[25]
*HIV co-infected*	0.032	Beta(0.2;13.1)	[0.000;0.123]	[25]
**Utilities**				
In untreated and uncured patients				
*F0-F3*	0.74		[0.718;0.767] ^b^	TAC trial [12]
*CC*	0.71		[0.687;0.732] ^b^	TAC trial [12, 34]
*DC*	0.66		[0.640;0.684] ^b^	TAC trial [12, 34]
*HCC*	0.66		[0.640;0.684] ^b^	TAC trial, [12, 35, 36]
During Treatment				
*F0-F3*	0.78		[0.763;0.807] ^b^	TAC trial [12]
*CC*	0.75		[0.729;0.771] ^b^	TAC trial [12, 34]
In cured patients				
*F0-F3*	0.81		[0.784;0.826] ^b^	TAC trial [12]
*CC*	0.77		[0.748;0.789] ^b^	TAC trial [12, 34]
*DC*	0.72		[0.698;0.736] ^b^	TAC trial [12, 34]
*HCC*	0.66		[0.640;0.684] ^b^	TAC trial [12, 35, 36]
**Health states costs**				
Health states costs with treatment at fibrosis stage				TAC trial [12] and micro-costing study
SOF/RBV				
Originator				
*Cameroon*	1660.1	Gamma(5790.7;0.3)	[1617.6;1703.1]	
*Cote d’Ivoire*	1439.7	Gamma(5790.7;0.2)	[1402.9;1477.0]	
*Senegal*	1570.7	Gamma(5790.7;0.3)	[1530.5;1611.4]	
Generic				
*Cameroon*	1029.8	Gamma(5790.7;0.2)	[1003.4;1056.5]	
*Cote d’Ivoire*	809.4	Gamma(5790.7;0.1)	[788.7;830.4]	
*Senegal*	940.4	Gamma(5790.7;0.2)	[916.3;964.8]	
SOF/LDV				
Originator				
*Cameroon*	1692.6	Gamma(5790.7;0.3)	[1649.3;1736.5]	
*Côte d’Ivoire*	1534.2	Gamma(5790.7;0.3)	[1494.9;1574.0]	
*Senegal*	1626.0	Gamma(5790.7;0.3)	[1584.4;1668.1]	
Generic				
*Cameroon*	920.4	Gamma(5790.7;0.2)	[896.8;944.3]	
*Côte d’Ivoire*	762.0	Gamma(5790.7;0.1)	[742.5;781.8]	
*Senegal*	853.8	Gamma(5790.7;0.1)	[831.9;875.9]	
SOF/DCV				
Originator				
*Cameroon*	1577.4	Gamma(5790.7;0.3)	[1537.0;1618.3]	
*Côte d’Ivoire*	1446.5	Gamma(5790.7;0.2)	[1409.5;1484.0]	
*Senegal*	1524.6	Gamma(5790.7;0.3)	[1485.6;1564.1]	
Generic				
*Cameroon*	521.4	Gamma(5790.7;0.1)	[508.1;534.9]	
*Côte d’Ivoire*	390.5	Gamma(5790.7;0.1)	[380.5;400.6]	
*Senegal*	468.6	Gamma(5790.7;0.1)	[456.6;480.7]	
SOF/VEL				
Originator				
*Cameroon*	1226.4	Gamma(5790.7;0.2)	[1195.0;1258.2]	
*Côte d’Ivoire*	1095.5	Gamma(5790.7;0.2)	[1067.5;1123.9]	
*Senegal*	1173.6	Gamma(5790.7;0.2)	[1143.6;1204.0]	
Generic				
*Cameroon*	776.4	Gamma(5790.7;0.1)	[756.5;796.5]	
*Côte d’Ivoire*	645.5	Gamma(5790.7;0.1)	[629.0;662.2]	
*Senegal*	723.6	Gamma(5790.7;0.1)	[705.1;742.4]	
Health states costs with treatment at CC stage				TAC trial [12] and micro-costing study
SOF/RBV				
Originator				
*Cameroon*	1841.3	Gamma(5790.7;0.3)	[1794.2;1889.0]	
*Côte d’Ivoire*	1628.8	Gamma(5790.7;0.3)	[1587.1;1671.0]	
*Senegal*	1790.6	Gamma(5790.7;0.3)	[1744.8;1837.0]	
Generic				
*Cameroon*	1211.0	Gamma(5790.7;0.2)	[1180.0;1242.4]	
*Côte d’Ivoire*	998.5	Gamma(5790.7;0.2)	[972.9;1024.4]	
*Senegal*	1160.3	Gamma(5790.7;0.2)	[1130.6;1190.4]	
SOF/LDV				
Originator				
*Cameroon*	1873.8	Gamma(5790.7;0.3)	[1825.8;1922.4]	
*Côte d’Ivoire*	1723.3	Gamma(5790.7;0.3)	[1679.2;1768.0]	
*Senegal*	1845.9	Gamma(5790.7;0.3)	[1798.7;1893.7]	
Generic				
*Cameroon*	1101.6	Gamma(5790.7;0.2)	[1073.4;1130.2]	
*Côte d’Ivoire*	951.1	Gamma(5790.7;0.2)	[926.8;975.8]	
*Senegal*	1073.7	Gamma(5790.7;0.2)	[1046.2;1101.5]	
SOF/DCV				
Originator				
*Cameroon*	1758.6	Gamma(5790.7;0.3)	[1713.6;1804.2]	
*Côte d’Ivoire*	1635.6	Gamma(5790.7;0.3)	[1593.7;1678.0]	
*Senegal*	1744.5	Gamma(5790.7;0.3)	[1699.9;1789.7]	
Generic				
*Cameroon*	702.6	Gamma(5790.7;0.1)	[684.6;720.8]	
*Côte d’Ivoire*	579.6	Gamma(5790.7;0.1)	[564.8;594.6]	
*Senegal*	688.5	Gamma(5790.7;0.1)	[670.9;706.3]	
SOF/VEL				
Originator				
*Cameroon*	1407.6	Gamma(5790.7;0.2)	[1371.6;1444.1]	
*Côte d’Ivoire*	1284.6	Gamma(5790.7;0.2)	[1251.7;1317.9]	
*Senegal*	1393.5	Gamma(5790.7;0.2)	[1357.8;1429.6]	
Generic				
*Cameroon*	957.6	Gamma(5790.7;0.2)	[933.1;982.4]	
*Côte d’Ivoire*	834.6	Gamma(5790.7;0.1)	[813.2;856.2]	
*Senegal*	943.5	Gamma(5790.7;0.2)	[919.4;968.0]	
Health states costs without treatment				TAC trial [12] and micro-costing study
F0-F3				
*Cameroon*	0	–	[0.0; 0.0]	
*Côte d’Ivoire*	0	–	[0.0; 0.0]	
*Senegal*	0	–	[0.0; 0.0]	
CC				
*Cameroon*	128.7	Gamma(658.5;0.2)	[119.1;138.7]	
*Côte d’Ivoire*	130.9	Gamma(658.5;0.2)	[121.1;141.1]	
*Senegal*	181.5	Gamma(658.5;0.3)	[167.9;195.6]	
DC				
*Cameroon*	143.2	Gamma(1380.2;0.1)	[135.7;150.9]	
*Côte d’Ivoire*	144.4	Gamma(1380.2;0.1)	[136.9;152.1]	
*Senegal*	194.0	Gamma(1380.2;0.1)	[183.9;204.4]	
HCC				
*Cameroon*	182.6	Gamma(399.6;0.5)	[165.1;200.9]	
*Côte d’Ivoire*	188.4	Gamma(399.6;0.5)	[170.4;207.3]	
*Senegal*	199.3	Gamma(399.6;0.5)	[180.2;219.3]	

*Abbreviations*: *CC* Compensated Cirrhosis, *CHC* Chronic Hepatitis C infection, *CI* Confidence Interval, *DC* Decompensated Cirrhosis, *F0, F1, F2, F3* METAVIR fibrosis stages, *SOF/DCV* Sofosbuvir/Daclatasvir, *SOF/LDV* Sofosbuvir/Ledipasvir, *SOF/RBV* Sofosbuvir/Ribavirin, *SOF/VEL* Sofosbuvir/Velpatasvir, *SVR12* Sustained Virologic Response measured at week 12 after treatment end

^a^95% CI calculated using the Wilson score formula.

^b^95% CI calculated using the bootstrap technique.

^c^The DSA considered alternative distributions: i) cohorts without CC; ii) cohorts with CC.

^d^Country, sex and age-specific

### Treatment strategies and comparator

Four 12-week sofosbuvir-based treatment regimens were assessed: i) sofosbuvir/ribavirin (genotype 2); ii) sofobuvir/ledipasvir (genotypes 1,4); iii) sofosbuvir/daclatasvir (genotypes 1,2,4); iv) sofosbuvir/velpatasvir (genotypes 1,2,4). The first two regimens - recommended in the WHO 2016 guidelines - were previously assessed in Cameroon, Côte d’Ivoire and Senegal in the ANRS-12311 TAC pilot trial [12]. Although no longer recommended in WHO 2018 guidelines, they are still being used in SSA. The two other regimens are recommended in the 2018 guidelines but have not yet been assessed in SSA.

With near-zero CHC treatment coverage in the study countries [2], we used the status-quo (i.e., no HCV treatment, whether DAA or Interferon-based) as a comparator.

### Outcomes

The main outcomes measured in the analysis were: i) Quality-adjusted life years (QALYs) (preferred in economic evaluations) [23], ii) costs estimated from the health system perspective, and iii) incremental cost-effectiveness ratios (ICERs).

### Model description

**Fig. 1:**
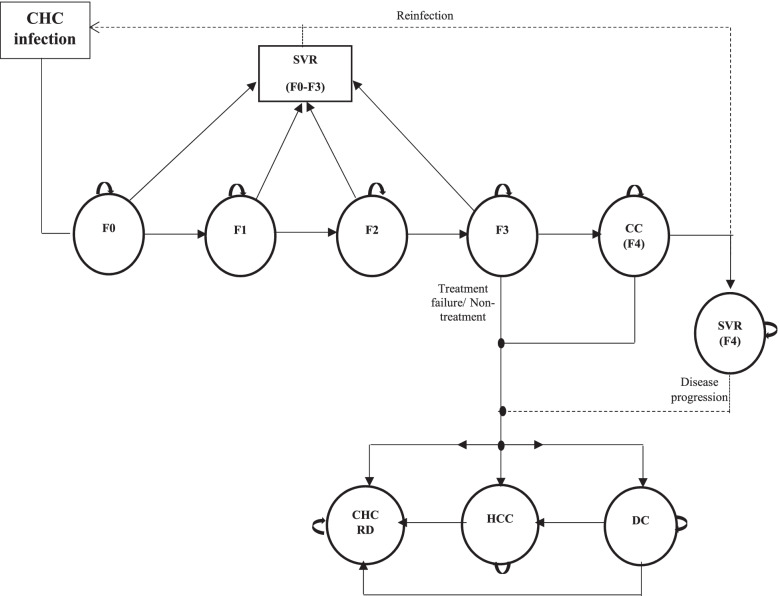
Simplified diagram of the Markov model. The oval boxes represent the different health states in the model, including two absorbing health states (CHC-related and CHC-unrelated deaths; the latter is not represented in the diagram for simplification purposes) and the following transient health states: fibrosis stages F0 to F3 (measured using the METAVIR scoring system), CC, DC and HCC. At model entry (CHC infection), all patients had a detectable viral load and were at the F0, F1, F2, F3 or CC stages. Arrows on full lines denote the transitions between health states according to treatment decision (i.e., whether patients received treatment or not) and treatment success (i.e., whether patients achieved SVR after treatment or not). The disease progression stops in all cured patients (i.e., who achieved SVR) except in patients in the CC health state at the end of the treatment cycle. Patients in the F3, CC, DC and HCC health states had a risk of CHC-related death. In addition, patients had a risk of CHC-unrelated death in all health states, corresponding to the “natural mortality” rate, which depends on age, gender and country. Arrows on dashed lines show reinfection in patients who achieved SVR. Abbreviation: CC, Compensated Cirrhosis; CHC, Chronic Hepatitis C; CHC RD, Chronic hepatitis C-related death; DC, Decompensated Cirrhosis; F0-F3, METAVIR fibrosis stages F0 to F3; HCC, Hepatocellular Carcinoma; SVR, Sustainable Virologic Response

The structure of the Markov model is depicted in Fig. 1. Markov models with a similar structure have been widely used in the HCV literature [17–19].

The initial distribution of patients in the different health states, defined according to the natural history of CHC (including fibrosis stage measured with the METAVIR scoring system F0 to F3, compensated cirrhosis (CC), decompensated cirrhosis (DC), and HCC), were as follows: 7.6% were in F0, 39.2% in F1, 26.2% in F2, 18.6% in F3 and 8.4% in CC [24].

Except for the first cycle, whose duration was set to 24 weeks (i.e., 12 weeks of treatment and 12 weeks of post-treatment follow-up), cycle durations were set to 1 year to reflect both the relatively slow progression of the disease and data availability. We assumed that transitions between health states occurred in the middle of the cycles using half-cycle corrections [22].

Disease progression over cycles depended on whether or not patients in the cohorts received sofosbuvir-based regimens and achieved SVR or not at the end of the treatment cycle.

In the untreated cohorts, all patients had active hepatitis with a persistently detectable VL, and progressed to more advanced disease stages according to the infection’ natural history. In the treated cohorts, all patients received sofosbuvir-based regimens in the first treatment cycle. We assumed that despite treatment, CHC could still progress to a more advanced disease stage during that cycle. At the end of the treatment cycle, patients either achieved SVR (cured) or did not (uncured). In subsequent cycles, we assumed that, apart from patients in the CC health state at the end of the treatment cycle, cured patients did not progress to a more advanced disease stage [1]. Furthermore, we made the conservative hypothesis that cured patients had a risk of reinfection in the subsequent cycles but were not newly treated [25], and had no liver fibrosis regression [26, 27]. Disease stage progression was similar for untreated patients and uncured patients.

### Model inputs

All parameter values and sources are presented in Table 1, while additional information is provided in Additional file 1.

#### DAA effectiveness

The effectiveness and safety of sofosbuvir/ribavirin and sofosbuvir/ledipasvir were estimated using data from the phase IIb, non-randomized TAC pilot trial (ANRS 12311), which was conducted from November 2015 to November 2017 in four hospitals in the study countries’ capital cities [12]. Briefly, 120 CHC patients mono-infected or HIV co-infected with HCV genotypes 1, 2 or 4, were treated over 12 weeks either with sofosbuvir/ribavirin (for genotype 2, *n* = 40) or sofosbuvir/ledipasvir (for genotypes 1 and 4, *n* = 40 in each group). Participants baseline characteristics were mostly similar in both treatment groups [12]. For each regimen, SVR was assessed 12 and 24 weeks after treatment end (SVR12 and SVR24). Results showed that 107/120 participants achieved SVR at both time points. SVR rates with and without cirrhosis were estimated at 0.793 [95% confidence intervals (CI): 0.705; 0.859] and 0.922 [0.820; 0.999] for sofosbuvir/ribavirin and 0.782 [0.716; 0.859] and 0.909 [0.832; 0.973] for sofosbuvir/ledispavir (See Table 1 and Additional file 1, p.3-6 for further details).

As no SVR data were available for pangenotypic regimens in SSA, SRV rates for sofosbuvir/daclatasvir and sofosbuvir/velpatasvir were estimated using data from the French national cohort HEPATER [28], adjusted for potentially lower treatment effectiveness in SAA (versus high-income countries) using TAC pilot trial data [12, 13]. SVR rates with and without cirrhosis were estimated at 0.824 [0.770; 0.870] and 0.958 [0.895; 1.000] for sofosbuvir/daclatasvir, and 0.840 [0.785; 0.887] and 0.977 [0.913; 1.000] for sofosbuvir/velpatasvir. 95% CI for all SVR rates were obtained by bootstrapping (See Table 1 and Additional file 1, p.21).

#### Utility scores

Face-to-face questionnaires administrated to participants in the TAC pilot trial at baseline, during treatment (i.e., at week 2 (W2), W4, W8 and W12), and then every 3 months until the end of follow-up (i.e., at W24 and W36) collected data on health-related quality of life (HRQoL) using the most recent version of the 12-item Short-Form survey (SF-12v2) [37].

Participants’ answers to the HRQoL questionnaire were used to classify them into one of the 18,000 health states described by the six-dimensional health state short form (SF-6D derived from the SF-12v2) [38]. A mapping algorithm - developed by the University of Sheffield using the standard gamble valuation technique - was then used to obtain the set of preference-based utility scores associated with each SF-6D health state [38].

As the model health states were defined according to the natural history of CHC (including fibrosis stage, CC and DC), we subsequently estimated the utility scores associated with each health states. More specifically, utility scores for F0 to F3 fibrosis health states before, during and after treatment were estimated as the mean utility scores for participants classified in these respective health states during follow-up. Corresponding 95% CI were obtained by bootstrapping. As few TAC trial participants were in the CC health state, and none in the DC and HCC states, we used additional data sources to estimate utility scores for these health states [34–36] (Additional file 1, p.6-9).

#### Transition probabilities

All other parameters used to simulate disease progression were derived from the literature, prioritizing data from SSA countries when available (Table 1 and Additional file 1, p.9-11 for further details).

#### Costs and DAA scenarios

The cost of the model's health states were estimated for each country using a micro-costing approach. The following costs items were included: laboratory tests, outpatient consultations, DAA treatment, concomitant drugs (for liver complications), inpatient and palliative care. The quantities of healthcare resources used according to the stages of CHC (F0-F3, CC and DC; with and without DAA) were defined based on the TAC pilot trial data complemented with hepatitis expert interviews in the study countries, in accordance with WHO 2018 guidelines (Additional file 1, p11-13) [16]. The respective unit costs of healthcare resources (except DAA) were obtained for the year 2017 using data collection in the study countries (Additional file 1, p13-15 and Table S4). For each model's health state and country, the total cost was computed as the sum of each healthcare resource used to care for patients in a given health state, multiplied by its respective unit cost (Additional file 1, p18 and Table S5).

In the base-case analysis, we considered two DAAs pricing scenarios: i) originator prices in the period when the TAC pilot trial was implemented (S1); ii) recent (2017) generic prices (S2). Prices for the two non-pangenotypic regimens were obtained from the WHO Global Price Reporting Mechanism database [39] for S1 and from WHO [40] for S2. In both scenarios, prices for the two pangenotypic regimens were obtained from Médecins Sans Frontières [20].

Respective DAA originator and generic prices for 12 weeks of treatment were as follows: US$1036 and US$406 for sofosbuvir/ribavirin, US$1200 and US$429 for sofobuvir/ledipasvir, US$1251 and US$195 for sofosbuvir/daclatasvir, and US$900 and US$450 for sofosbuvir/velpatasvir.

Costs in *Franc de la Communauté Financière Africaine* were converted to US dollars using year-specific exchange rates and expressed in 2017 US dollars [41].

### Economic and sensitivity analysis

The economic analysis was performed according to international guidelines [42, 43]. For the status-quo and for each regimen we estimated the following: i) the expected lifetime costs per patient and ii) expected lifetime health benefits per patient assessed in terms of QALYs (computed as the time spent in a specific health state weighted by the utility score corresponding to that health state). Options were then ordered by growing lifetime costs and ICERs were computed for each non-dominated regimen, compared with the next best alternative, as the incremental expected lifetime cost per patient divided by the incremental expected lifetime health benefit per patient. ICERs were then compared with the country’s cost-effectiveness threshold (CET) to assess the cost-effectiveness. Given the strong constraints on the healthcare system’s budget in the three study countries, we assumed a CET of 0.5 times the 2017 country’s per-capita gross domestic product (GDP) based on the opportunity cost approach [44] (i.e., US$711 in Cameroon, US$778.5 in Côte d’Ivoire and US$683.5 in Senegal [45]). Furthermore, because of the uncertainty around the value of the CET, we used the cost-effectiveness acceptability curve (CEAC) to indicate the probability of the preferred regimens being cost-effective for lower CET.

We addressed uncertainty in the model parameters using a probabilistic sensitivity analysis (PSA) with Monte Carlo simulations including 10,000 iterations [46]. Model parameter values were randomly drawn from their predefined distributions to obtain 10,000 simulated pairs of incremental expected lifetime costs and QALYs. Based on standard practice [46], transition probabilities and utility scores parameters were assumed to follow a Beta distribution and costs were assumed to follow a Gamma distribution (Table 1 and Additional file 1, p.21-25). The simulated pairs were plotted in the cost-effectiveness plane, and their respective distributions used to compute incremental expected lifetime costs and QALYs with corresponding 95% CI. We also used the Monte Carlo simulations to calculate the probability of the preferred regimens being cost-effective at various CET and to derive the corresponding CEAC by plotting these probabilities on the y-axis versus the CET on the x-axis.

Furthermore, as we had a large number of parameters in the model, we identified those which contributed the most to the variability of the cost-effectiveness results (i.e., with the highest R-squared value) using linear regression models. Finally, a Deterministic Sensitivity Analysis (DSA) was performed for the most expensive lifetime treatment regimen for each pricing scenario. First, we varied the discount rate at 0, 3, and 5%. Second, to assess the impact of initiating DAA at earlier/later disease stages, we simulated cohorts with no/all patient in the CC health state at model entry. Third, we simulated cohorts whose risk of HCV reinfection was higher than in the base-case [25].

The model’s internal validity was tested. The methods used and consequent results are reported in Additional file 1, p.25. All analyses were performed using R [47], version 3.6.0 (m*arkovchain* package*)* [48].

## Results

### Base-case analysis

Expected lifetime costs, QALYs and ICERs are presented for each country in Table 2.

**Table 2:** Expected lifetime Quality adjusted life-years (QALYs), Costs (US dollars 2017) and incremental cost-effectiveness ratios (ICERs) of Sofosbuvir-based regimens in Cameroon, Côte d’Ivoire and Senegal (each modeled cohort = 10,000 mono-infected patients)

	QALYs (mean [95%CI]), per patient			Costs (mean [95%CI]), per patient (2017 USD)			ICERs (mean [95%CI]), per QALY			Probability of being CE^a^		
	Cam.	Côte d’Iv.	Sen.	Cam.	Côte d’Iv.	Sen.	Cam.	Côte d’Iv.	Sen.	Cam.	Côte d’Iv.	Sen.
**Scenario 1: originator DAA prices**												
Status-quo	8.4 [8.1; 8.8]	7.9 [7.6; 8.3]	8.7 [8.3; 9.1]	274.8 [218.5; 336.8]	256.0 [202.6; 311.8]	387.1 [305.4; 473.3]	–	–	–	–	–	–
SOF/ DCV	10.1 [9.9; 10.4]	9.4 [9.2; 9.7]	10.5 [10.2; 10.8]	1695.1 [1651.7; 1738.1]	1560.6 [1520.3; 1600.4]	1689.4 [1645.5; 1735.7]	Dominated	Dominated	Dominated			
SOF/ VEL	10.2 [9.9; 10.4]	9.5 [9.2; 9.7]	10.6 [10.3; 10.8]	1340.7 [1306.2; 1376.3]	1206.9 [1176.0; 1239.0]	1334.6 [1297.2; 1373.3]	621.0 [481.5; 819.2]	632.0 [483.1; 850.5]	525.6 [402.5; 704.9]	0.86	0.93	0.96
SOF/ RBV	10.1 [9.8; 10.4]	9.4 [9.1; 9.6]	10.5 [10.1; 10.8]	1783.3 [1735.6; 1832.3]	1559.5 [1518.1; 1602.0]	1743.9 [1695.2; 1795.9]	Dominated	Dominated	Dominated			
SOF/ LDV	10.1 [9.8; 10.3]	9.4 [9.1; 9.6]	10.4 [10.2; 10.7]	1818.1 [1771.3; 1866.7]	1655.3 [1613.3; 1698.6]	1802.2 [1753.0; 1853.2]	Dominated	Dominated	Dominated			
**Scenario 2**: **generic DAA prices**												
Status-quo	8.4 [8.1; 8.8]	7.9 [7.6; 8.3]	8.7 [8.3; 9.1]	274.8 [218.5; 336.8]	256.0 [202.6; 311.8]	387.1 [305.4; 473.3]	–	–	–	–	–	–
SOF/ DCV	10.1 [9.9; 10.4]	9.4 [9.2; 9.7]	10.5 [10.2; 10.8]	639.0 [618.3; 661.2]	504.5 [487.2; 523.3]	633.5 [607.9; 661.8]	215.8 [157.2; 297.2]	168.3 [117.1; 237.5]	139.4 [85.9; 208.4]	1	1	1
SOF/ VEL	10.2 [9.9; 10.4]	9.5 [9.2; 9.7]	10.6 [10.3; 10.8]	891.2 [866.5; 917.2]	757.0 [735.2; 779.6]	885.0 [856.6; 916.5]	2522 [2483; 5104]	2525 [2480; 6427]	2515 [2487-5073]	0	0	0
SOF/ LDV	10.1 [9.8; 10.3]	9.4 [9.1; 9.6]	10.4 [10.2; 10.7]	1046.1 [1016.3; 1077.4]	882.9 [857.0; 909.6]	1029.7 [995.9; 1066.0]	Dominated	Dominated	Dominated			
SOF/ RBV	10.1 [9.8; 10.4]	9.4 [9.1; 9.6]	10.5 [10.2; 10.8]	1153.3 [1120.7; 1187.7]	928.8 [901.6; 958.8]	1113.4 [1076.5; 1154.4]	Dominated	Dominated	Dominated			

*Abbreviations*: *Cam.* Cameroon, *CE* Cost-effective, *Côte d’Iv.* Côte d’Ivoire, *ICER* Incremental cost-effectiveness ratio, *QALYs* quality adjusted life-years, *Sen.* Senegal, *SOF/DCV* Sofosbuvir+Daclatasvir, *SOF/LDV* Sofosbuvir+Ledipasvir, *SOF/RBV* Sofosbuvir + Ribavirin, *SOF/VEL* Sofosbuvir+Velpatasvir

^a^At a cost-effectiveness threshold of 0.5 times the country per-capita gross domestic product

Without treatment, the expected lifetime costs [95% CI] per patient ranged from US$256 [203;312] to US$387 [305;473], depending on the country. In S1, sofosbuvir/velpatasvir had the lowest expected lifetime costs (estimated between US$1207 [95% CI: 1176;1239] and US$1341 [1306;1376] per patient). The lifetime expected costs per patient of the three other regimens were not significantly different, ranging from US$1560 [1518;1602] for sofosbuvir/ribavirin in Côte d’Ivoire to US$1818 [1771;1867] for sofosbuvir/ledipasvir in Cameroon.

In S2, expected lifetime costs per patient decreased between 58 and 67%, except for sofosbuvir/daclatasvir - where expected lifetime costs were 2.6 to 3 times lower than in S1. This regimen had the lowest expected lifetime costs (range of costs [95% CI]: US$505 [487;523] to US$639 [618;661]). Sofosbuvir/velpatasvir was the second least costly regimen (range of costs [95% CI]: US$757 [735;780] to US$891 [867;917]).

Expected lifetime QALYs [95% CI] per patient ranged from 7.9 [7.6;8.3] to 8.7 [8.3;9.1] without treatment and 9.4 [9.1;9.6] to 10.6 [10.3;10.8] with treatment. Health benefits were slightly but not significantly higher for sofosbuvir/velpatasvir, while the three other regimens had very similar health benefits.

**Fig. 2:**
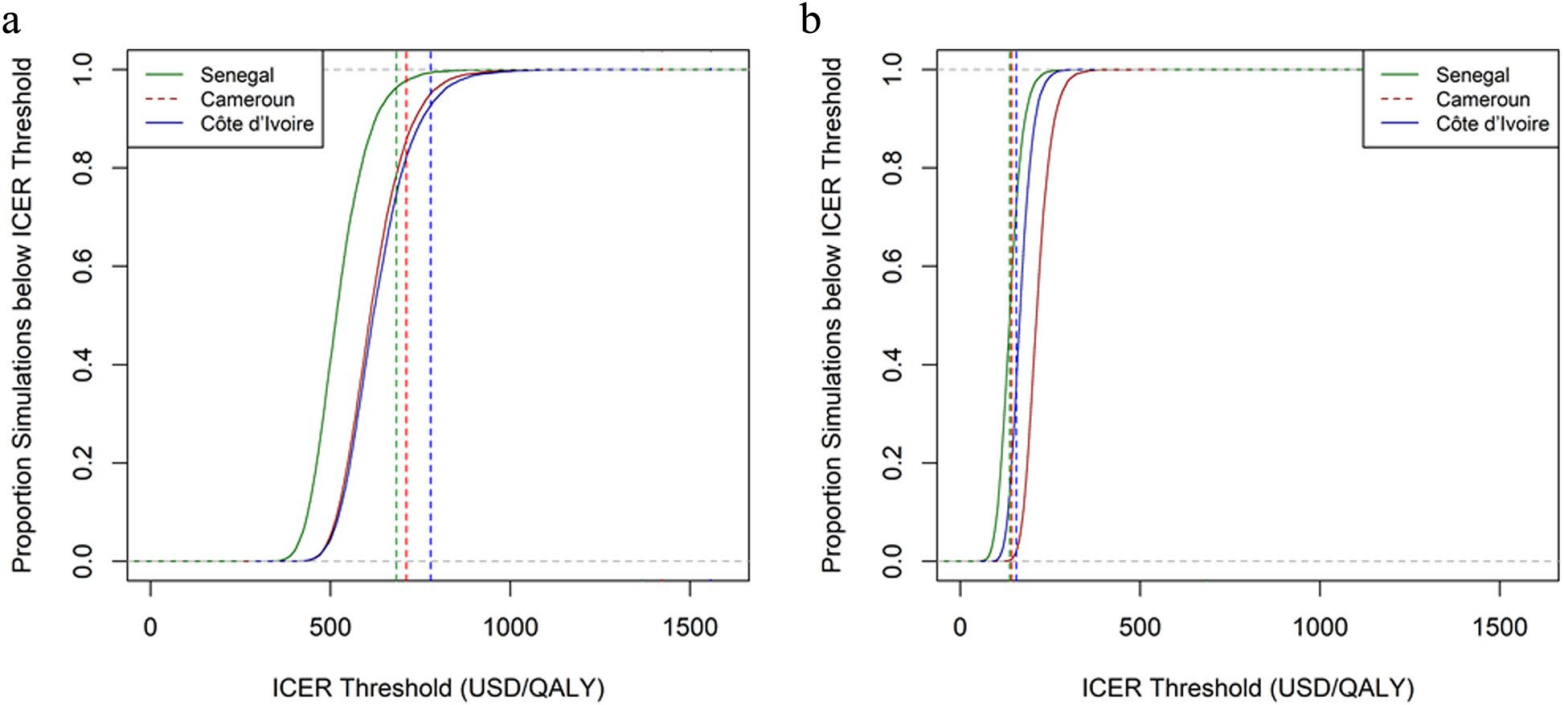
Cost-effectiveness acceptability curves for sofosbuvir/velpatasvir versus the status-quo (Scenario 1: originator prices) (**a**) and for sofosbuvir/daclatasvir versus the status-quo (Scenario 2: generic prices) (**b**) in Cameroon, Côte d’Ivoire and Senegal. The colored vertical lines (green, red and blue) indicate the cost-effectiveness thresholds of 0.5 times the GDP/capita in 2017 for each of the three study countries (i.e., US$683.5 in Senegal, US$711 in Cameroon, US$778.5 in Côte d’Ivoire, respectively). The cost-effectiveness acceptability curves show the probability that the preferred regimen in each scenario (i.e., sofosbuvir/velpatasvir in scenario 1 (**a**) and sofosbuvir/daclatasvir in scenario 2 (**b**)) is cost-effective compared with the status quo at various cost-effectiveness thresholds ranging from US$0 to US$1500/QALY. The green, red and blue curves correspond, respectively, to Senegal, Cameroon and Côte d’Ivoire. Abbreviations: GDP: Gross Domestic Product; ICER: Incremental cost-effectiveness ratio; QALYs: Quality adjusted Life-years

In S1, sofosbuvir/velpatasvir dominated the three other regimens as it had significantly lower costs but slightly better health benefits; accordingly, it was the preferred DAA regimen. At a CET of 0.5 times the country’s per-capita GDP, sofosbuvir/velpatasvir was cost-effective with a probability of 86, 93 and 96% in Cameroon, Côte d’Ivoire and Senegal, respectively. As illustrated by the CEAC (Fig. 2), the probability of sofosbuvir/velpatasvir being cost-effective was > 95% for a CET higher than US$777, US$805 and US$666 in Cameroon, Côte d’Ivoire and Senegal, respectively, which is slightly above the CET of 0.5 times the country’s CET (except for Senegal).

In S2, sofosbuvir/ribavirin and sofosbovir/ledispavir were dominated by both sofosbuvir/daclatasvir and sofosbuvir/velpatasvir, as they were significantly less costly with similar or slightly higher health benefits. ICERs [95% CI] ranged from US$139 [86; 208] per QALY (Senegal) and US$216 [157; 297] per QALY (Cameroon) for sofosbuvir/daclatasvir compared with the status-quo, and from US$2515 [2487; 5073] per QALY (Senegal) to US$2525 [2487; 5073] per QALY (Côte d’Ivoire) for sofosbuvir/velpatasvir compared with sofosbuvir/daclatasvir. At the CET of 0.5 times the country’s per-capita GDP, sofosbuvir/daclatasvir had a 100% probability of being cost-effective. In addition, the CEAC (Fig. 2) showed that sofosbuvir/daclatasvir had a > 95% probability of being cost-effective for all CET higher than US$281/QALY, US$223/QALY and US$195/QALY in Cameroon, Côte d’Ivoire and Senegal, respectively, which corresponded to 0.14 (Côte d’Ivoire and Senegal) and 0.2 (Cameroon) times the corresponding country’s per-capita GDP. At a CET of 0.5 times the country per-capita GDP, sofosbuvir/velpatasvir was not cost-effective when compared with sofosbuvir/daclatasvir.

The model parameters with the largest contributions to the variability of the cost-effectiveness results were the utility scores (for all health states), the transition probabilities (from F3 to CC and from F2 to F3 in untreated and uncured patients) and the SVR rate (Supplemental Table S6).

### Deterministic sensitivity analysis (DSA)

Results from the DSA conducted for the most expensive lifetime treatment strategy in both scenarios (i.e., sofosbuvir/daclatasvir in S1 and sofosbuvir/velpatasvir in S2) are presented in Supplemental Fig. S2.

In S1, the DSA showed that the sofosbuvir-based regimens were not cost-effective (probability > 95%) for all parameter variations at the CET of 0.5 times the per-capita GDP, with the exception of the scenario where the discount rate was 0% because of the long-term health benefits of treatment while costs mainly occurred in the short term.

By contrast, in S2, the sofosbuvir-based regimens remained cost-effective (with a probability> 95%) for most of the various parameters’ variations (at a CET of 0.5 times the per-capita GDP). More specifically, the cost-effectiveness improved in most scenarios except in the following three scenarios. First, when increasing the discount rate to 5%, ICERs slightly increased but the probability of being cost-effective remained 100% in each country. Second, when assuming that all patients were at the CC stage at model entry, ICERs strongly increased because of the much lower health benefits (due to the lower SVR rates in patients at the CC stage and the residual risk of progressing to stages DC and HCC despite HCV cure) and sofosbuvir-based regimens were no longer cost-effective. Third, a higher reinfection risk led to a slight increase in ICERs because of lower health benefits than in the base-case. However, sofosbuvir-based regimens remained cost-effective with a probability > 95%.

## Discussion

This is the first study to assess the cost-effectiveness of DAA for the treatment of CHC in SSA.

In S1 at originator prices, sofosbuvir/velpatasvir was the preferred DAA regimen. However, at a CET of 0.5 times the country’s per-capita GDP, this regimen was only cost-effective in Senegal (with a probability > 95%) and was not cost-effective when considering lower CET. In S2 at generic prices, sofosbuvir/daclatasvir provided the best value for money (ICERs range: US$139-216/QALY according to country) due to significantly lower lifetime costs. Generic sofosbuvir/daclatasvir was cost-effective for all CET higher than US$281/QALY, US$223/QALY and US$195/QALY in Cameroon, Côte d’Ivoire and Senegal, respectively, corresponding to 0.14 (Côte d’Ivoire and Senegal) and 0.2 (Cameroon) times the country’s per-capita GDP. In countries with serious resource constraints, funding interventions based on such a low CET would bring additional health benefits at the population level as their cost/QALY is lower than that of other funded interventions like HIV treatment [44]. The DSA also suggested that sofosbuvir-based regimens were more cost-effective when initiated early (i.e., at mild fibrosis stage) but not cost-effective when initiated at the cirrhosis stage. This finding highlights the importance of early diagnosis.

The lowest lifetime costs observed for both pangenotypic regimens were partly driven by the absence of the need for laboratory-based genotyping before treatment initiation. Furthermore, in S1, sofosbuvir/velpatasvir had lower lifetime costs than sofosbuvir/daclatasvir because of lower DAA prices. This may be explained by the availability of a fixed-dose combination of sofosbuvir/velpatasvir resulting in lower production and marketing costs. Conversely, as the generic version of sofosbuvir/daclatasvir can also be produced in a fixed-dose combination, it cost approximately twice as less as sofosbuvir/velpatasvir in S2 [20] making it the regimen with the lowest cost.

Although sofosbuvir/velpasvir tended to have slightly higher QALYs, we found relatively similar lifetime health benefits in terms of QALYs for all four regimens. This is somewhat surprising, as higher SVR rates have been reported for pangenotypic regimens [49], but may be explained by our conservative approach when estimating SVR rates for sofosbuvir/daclatasvir and sofosbuvir/velpatasvir. Indeed, recent studies found that specific genotype 4 subtypes, such as genotype 4r, may lead to lower DAA effectiveness in SSA [12, 13]. Although in-vitro studies have suggested that sofosbuvir/velpatasvir could potentially be more effective than sofosbuvir/daclatasvir [50], no evidence exists to date for the effectiveness of pangenotypic regimens in SSA.

Many studies, mostly conducted in high and upper-middle income countries, have demonstrated the economic value of DAA [17–19, 51]. Only one study has reported data on the cost-effectiveness of DAA in a low-income setting, specifically in Cambodia [52]. However, the study’s results were limited by the intervention’s specific setting, which differed from real-world healthcare delivery in national health system. Our study - performed in a setting near real-world healthcare delivery in SAA - demonstrates for the first time that at current generic prices, using pangenotypic DAAs, and more specifically sofosbuvir/daclatasvir to treat chronic hepatitis C, is a cost-effective intervention which deserves to be funded.

Despite the strong evidence we provide here for the value for money of DAA in SSA, the real-world scaling-up of this treatment raises two key questions: i) To what extent are generic drugs already realistically available in these countries, ii) Will national governments and patients be able to afford them?

With regard to availability, while the importation and manufacture of generic DAA is theoretically now possible, two important regulatory steps are required before DAA - generic or originator - can be sold in a country: WHO prequalification and market authorization by national authorities.

In our three study countries, only sofosbuvir and daclatasvir have WHO prequalification for both the originator and generic versions. Only the originator’s version of velpatasvir has been prequalified. No prequalification exists for ledipasvir. With regard to drug registration by national authorities, at least three generic manufacturers are already registered for daclatasvir in Cameroon, while at least one has applied for market authorization in Côte d’Ivoire and Senegal [53]. Generic versions of Gilead’s drugs are registered in Cameroon and Côte d’Ivoire but we found no information for Senegal.

These data suggest that sofosbuvir/daclatasvir seems to be the pangenotypic regimen most likely to become widely available in generic versions in the three study countries, and probably in other SSA countries. Moreover, a study by Van de Ven et al. suggested that daclatasvir is particularly inexpensive to produce, and that the economies of scale which might be achieved with the scaling-up of DAA, could reduce the cost of profitably of mass producing a 12-week course of the generic version of daclatasvir to US$76 [54]. This strongly suggests that producing and using generic DAA must be a core strategy to effectively reach the WHO’s target of global HCV elimination by 2030.

With regard to affordability, we estimated the mean total cost for treating CHC with generics sofosbuvir/daclatasvir in the study countries at US$406-537 (including treatment, consultations and biological tests). Out-of-pocket payments are the prevalent financing modality of the study countries’ health systems, meaning that if the whole cost of CHC treatment is borne by patients, it is likely to lead to unmet needs and catastrophic health expenditures.

If instead governments were to bear the total cost of CHC alone, treating all CHC patients currently diagnosed with generic sofosbuvir/daclatasvir would cost US$1.7 million in Cameroon, US$3.9 million in Côte d’Ivoire, and US$3 million in Senegal. Furthermore, in order to reach the WHO’s target of treating 80% of CHC patients by 2030 [2], the costs involved would increase substantially to US$37.8, US$53.8 and US$42.1 million, respectively (or 19.4, 12.8, 15.6% of each government’s current health expenditure).

This suggests that a substantial increase in both national health expenditures and international funding would be required to meet the WHO’s targets for 2030, unless current health expenditures were reallocated to provide CHC treatment and further decreases in generic prices were obtained for DAA treatments.

Our study has limitations. First, as there is no evidence for the effectiveness of pangenotypic regimens in SSA, we estimated related SVR rates using data from a high income setting (HEPATER cohort, France) [28]. However, we used conservative estimates in the base-case analysis and identified SVR thresholds for which DAAs had a > 95% probability of being cost-effective (54 and 70% for sofosbuvir/velpatasvir and sofosbuvir/daclastasvir, respectively).

Second, due to the lack of natural history data in SSA, the transition probabilities used to model disease progression were primarily obtained from studies conducted in high-income settings. In SSA, where access to specialized care for liver complications is limited, disease progression and mortality may occur faster. This may have resulted in an underestimation of mortality in the absence of treatment, and consequently, in an underestimation of the benefits of DAA. However, unlike CHC patients living in high-income countries, CHC patients in SSA may have lower hepatic comorbidities (i.e., liver disease (whether alcohol-related or not)), resulting in lower morbidity and mortality. To mitigate the effect of uncertainty on the model’s parameters, we performed probabilistic sensitivity analysis in line with international standards [46].

Third, our study was conducted in only three countries in West and Central Africa. Despite this, we believe that our findings may be generalizable to other countries of SSA, as cost-effectiveness is highly dependent on DAA prices that are set by international agreements, and are therefore relatively similar across countries with comparable living standards.

## Conclusion

Our study demonstrates that the use of generic pangenotypic sofosbuvir-based regimens for the treatment of chronic hepatitis C provided good value for money in SSA. More specifically, when considering generic DAA currently available in SSA and their current prices, sofosbuvir/daclatasvir was the preferred option. Large-scale use of generics to obtain further reductions in DAA prices, and an increase in national and international funding for hepatitis C treatment, must be priorities for the HCV elimination agenda.

## Supplementary Information

**Additional file 1.**

## Data Availability

Detailed information on data used are provided in the Supplementary information file. The datasets used during the current study are available from the corresponding author on reasonable request.

## Abbreviations

CC: Compensated cirrhosis
CET: Cost-effectiveness threshold
CHC: Chronic hepatitis C
CI: Confidence intervals
DAA: Direct-acting antivirals
DC: Decompensated cirrhosis
F0, F1, F2, F3: METAVIR fibrosis stages
HCC: Hepatocellular carcinoma
HCV: Hepatitis C virus
ICER: Incremental cost-effectiveness ratio
QALY: Quality-adjusted life years
SAA: Sub-Saharan Africa
SVR: Sustained virologic response
WHO: World Health Organization
