# Supplementary material for: Cost-utility analysis of four WHO-recommended sofosbuvir-based regimens for the treatment of chronic hepatitis C in sub-Saharan Africa
Source: BMC Health Serv Res. 2022 Mar 5;22:303. doi: 10.1186/s12913-021-07289-0 (PMC8897946; doi:10.1186/s12913-021-07289-0)
Supplement: Supplementary file 1 — Additional file 1. [file 12913_2021_7289_MOESM1_ESM.docx]

**Additional file 1**

This Additional file 1 is provided by the authors to give the reader additional information about the work performed.

Supplement to: **Cost-utility analysis of four WHO-recommended sofosbuvir-based regimens for the treatment of chronic hepatitis C in sub-Saharan Africa**

**Contents:**

Page 3-22: Technical documentation on the model

- Pages 3-17: Model inputs
  - Page 5: Table S1. Overall SVRs rates of sofosbuvir-based regimens in the TAC pilot trial and in the HEPATHER cohort
  - Page 6: Table S2. SVRs rates of sofosbuvir-based regimens in the TAC pilot trial and in the HEPATHER cohort with and without cirrhosis
  - Page 8: Table S3. Utilities scores associated with model health states
  - Pages 16-17: Table S4. Unit costs per country (US dollars, 2017) and annual quantities of healthcare resources associated with model health states
  - Pages 19-20: Table S5: Model health state costs (in US dollars, 2017)
- Pages 21-25: Probabilistic sensitivity analysis
- Page 25: Model validity

Pages 26-27: Table S6. Model parameter contributions to the variability of cost-effectiveness results

Pages 28-30: Figure S2. Deterministic sensitivity analysis for Scenario 1 (originator prices) (Fig. S2a) and Scenario 2 (generic prices) (Fig. S2b)

Pages 31-33: References

**Technical documentation on the model**

1. **Model inputs**
   1. ***Effectiveness of DAAs***
2. *Overall effectiveness of non-pangenotypic treatment (i.e., sofosbuvir/ribavirin and sofosbuvir/ledipasvir)*

The effectiveness of sofosbuvir/ribavirin (SOF/RBV) and sofosbuvir/ledipasvir (SOF/LDV) regimens was estimated using data from the TAC pilot trial. For each treatment strategy, we estimated the respective mean (95% CI) SVR rate, assessed 12 weeks after the end of treatment, for all trial participants (n=120).

1. *Overall effectiveness of pangenotypic treatments (i.e., sofosbuvir/daclatasvir and sofosbuvir/velpatasvir)*

As the effectiveness of sofosbuvir/daclatasvir (SOF/DCV) and sofosbuvir/velpatasvir (SOF/VEL) were not assessed within the TAC trial or in other studies conducted in the sub-Saharan Africa (SSA) setting, we used data from the French national ANRS CO22 HEPATHER cohort^1^ to estimate the SVR of SOF/DCV and SOF/VEL. This ongoing French nationwide multicenter cohort (ClinicalTrials.gov, number NCT01953458) started in 2012. It enrolled adult individuals (aged ≥18 years), with active or inactive hepatitis B virus (HBV) infection (with or without hepatitis D virus (HDV) infection) and/or hepatitis C virus (HCV) infection (whether cured or not). Detailed information about the cohort design has been published elsewhere^1,2^.

Given that the SVR rates of SOF/RBV and SOF/LDV obtained in the TAC trial were lower than those found in HEPATHER and in other trials conducted in high income countries^3–7^, we adjusted downwards the SVR rates of SOF/DCV and SOF/VEL estimated in the HEPATHER cohort to take into account the potentially lower effectiveness of pangenotypic regimens in SSA. We assumed that the differences observed between non-pangenotypic regimen SRV rates assessed in the TAC trial and in the HEPATHER cohort (see Table S1 page 3), would be the same for SOF/DCV and SOF/VEL SVR, as follows:

$SVR\left( DCV \right)_{TAC}= SVR\left( DCV \right)_{HEPATHER}\times\frac{SVR\left( overall \right)_{TAC}}{SVR\left( overall \right)_{HEPATHER}}$ where $SVR\left( overall \right)_{HEPATHER}$ is calculated as the mean SVR rate of SOF/RBV and SOF/LDV observed in the HEPATHER cohort weighted by the proportion of patients receiving each of the two non-pangenotypic regimens in the TAC clinical trial.

Finally, $SVR\left( DCV \right)_{TAC}=0.962 \times\frac{0.892}{\left( \frac{1}{3}\times0.802+ \frac{2}{3}\times0.975 \right)}=0.935$.

The same correction was applied to derive confidence intervals.

Similarly, the SVR rate for the SOF/VEL regimen was calculated as follows:

$$SVR\left( VEL \right)_{TAC}= SVR\left( VEL \right)_{HEPATHER}\times\frac{SVR\left( overall \right)_{TAC}}{SVR\left( overall \right)_{HEPATHER}}=0.981 \times\frac{0.892}{\left( \frac{1}{3}\times0.802+ \frac{2}{3}\times0.975 \right)}=0.953$$

The SVR rates for SOF/RBV and SOF/LDV estimated in the TAC trial and in the HEPATHER cohort, as well as the SVR rates for SOF/DCV and SOF/VEL before and after adjustment, are presented in supplemental Table S1 below.

**Table S1: Overall SVR rates of sofosbuvir-based regimens in the TAC pilot trial and in the HEPATHER cohort**

| **TAC trial** | | | **HEPATHER** | | | | | **In the study  (after adjustment)** | |
| --- | --- | --- | --- | --- | --- | --- | --- | --- | --- |
| SVR | SOF/  RBV | SOF/  LDV | Overall | SOF/ RBV | SOF/ LDV | SOF/ DCV | SOF/ VEL | SOF/ DCV | SOF/ VEL |
| N | 40 | 80 | 4618 | 485 | 2121 | 1557 | 368 | 10,000 | 10,000 |
| % | 0.900 | 0.888 | 0.952 | 0.802 | 0.975 | 0.962 | 0.981 | 0.935 | 0.953 |

Abbreviations: SOF/DCV: Sofosbuvir/Daclatasvir; SOF/LDV: Sofosbuvir/Ledipasvir; SOF/RBV: Sofosbuvir/Ribavirin; SOF/VEL: Sofosbuvir/Velpatasvir; SVR: Sustained Virologic Response measured at week 12 after treatment end.

1. *Effectiveness of DAAs in cirrhotic and non-cirrhotic patients*

Using the TAC trial data, we computed the following two ratios of SVR rates with and without cirrhosis over the overall SVR rate (for information: cirrhosis was assessed using the APRI score):

$Ratio_{no cirrhosis}= \frac{SVR\left( overall in patients with APRI score \leq2 \right)_{TAC}}{SVR\left( overall \right)_{TAC}}= \frac{0.786}{0.892}=0.881$

$Ratio_{cirrhosis}= \frac{SVR\left( overall in patients with APRI score > 2 \right)_{TAC}}{SVR\left( overall \right)_{TAC}}= \frac{0.913}{0.892}=1.024$

We then obtained the SVR rates in patients with cirrhosis and without cirrhosis, respectively, by multiplying these ratios by the SVR rate for each regimen obtained previously (i.e., SOF/RBV, SOF/LDV, SOF/DCV, SOF/VEL) (See Table S2 page 6).

**Table S2: SVR rates of sofosbuvir-based regimens in the TAC pilot trial and in the HEPATHER cohort with and without cirrhosis**

|  | **SOF/RBV** | **SOF/LDV** | **SOF/DCV** | | **SOF/VEL** | |
| --- | --- | --- | --- | --- | --- | --- |
|  | SVR rates in TAC (n=40) | SVR rates in TAC (n=80) | SVR rates in HEPATHER (before adjustment)  (n=4618) | SVR rates in the study (after adjustment) (n=10,000)^#^ | SVR rates in HEPATHER (before adjustment) (n=4618) | SVR rates in the study (after adjustment (n=10,000) ^#^ |
| Overall | 0.900 | 0.888 | 0.962 | 0.935 | 0.981 | 0.953 |
| Without cirrhosis | 0.922 | 0.909 | - | 0.958 | - | 0.977 |
| With cirrhosis | 0.793 | 0.782 | - | 0.824 | - | 0.840 |

^#^ Fictive cohorts.

Abbreviations: SOF/DCV: Sofosbuvir/Daclatasvir; SOF/LDV: Sofosbuvir/Ledipasvir; SOF/RBV: Sofosbuvir/Ribavirin; SOF/VEL: Sofosbuvir/Velpatasvir; SVR: Sustained Virologic Response measured at week 12 after treatment end.

95% Confidence Intervals (CI) for all SVR rates were obtained by bootstrapping using data from the TAC pilot trial (see subsection 2.3. p.23).

- 1. ***Utility scores***

HRQoL was assessed in the TAC trial participants using the most recent version of the 12-item Short-Form survey (SF-12v2)^8^ administrated at baseline, during treatment (at W2, W4, W8 and W12) and every 3 months until the end of follow-up (i.e., at W24 and W36).

Using this scale, participants were uniquely classified according to the six-dimensional health state short form (SF-6D) which describes 18,000 health states^9^. This generic preference-based single index measure of health can be used to generate QALYs for use in cost-utility analyses. Using a mapping algorithm developed by the University of Sheffield, a utility (or preference) score was attributed to each of the SF-6D health states^10^. These utility scores were obtained from a sample of the general population in the United Kingdom using the standard gamble valuation technique.

At the time of the study, the SF-6D utility scores were only available for a limited number of high-income countries (Australia, Brazil, Honk Kong, Japan, Portugal and Singapore). No SF-6D utility scores were available in a cultural, social and medical setting similar to those of the three study countries.

Utility scores related to the F0 to F3 health states (before, during and after treatment) were estimated as the mean utility scores of participants classified in these respective health states over follow-up. 95% CI were obtained using bootstrapping. The following estimations in patients in F0 to F3 health states were found: mean [95% CI] utility scores were 0.74 [0.72; 0.77] at baseline (i.e., in untreated and uncured patients), 0.78 [0.76; 0.81] during treatment (at W8) and 0.81 [0.78; 0.83] 12 weeks after the end of treatment (i.e., in cured patients). Utility scores were stable during the treatment and post-treatment period, respectively, with mean values of 0.79 at W2 and 0.78 at W4, W8 and W12 (treatment period), and 0.81 and 0.79 at W24 and W36, respectively (post-treatment period).

As there was a small number of TAC pilot trial participants in the compensated cirrhosis (CC) health state and none in the decompensated cirrhosis DC or hepatocellular carcinoma (HCC) health states, we used additional data sources to estimate related utility scores^11,12,13^.

First, we calculated the loss in utility score at the CC and DC stages compared with the fibrosis stage using the data from Stepanova et al.^11^ and multiplied the F3 health state’s utility score (estimated using the TAC trial data) by this loss in utility score. More specifically, Stepanova et al. reported the following mean utility scores (obtained using the SF-6D): 0.725, 0.693 and 0.646, for patients without cirrhosis, with compensated cirrhosis and with decompensated cirrhosis, respectively. Using these data, we computed two ratios: the CC utility score over the utility without cirrhosis, and similarly the DC utility score over the utility without cirrhosis, as follows:

$Ratio_{U(CC)/U(No Cirrhosis)}= \frac{0.693}{0.725}=0.956$ and $Ratio_{U(DC)/U(No Cirrhosis)}= \frac{0.646}{0.725}=0.891$

Utility scores measured in the TAC pilot trial at baseline (i.e., in patients with detectable viral load), during treatment and after treatment (i.e., in cured patients), were then multiplied by these ratios to derive the corresponding utility scores for patients in the CC and DC health states, respectively (see Table S3).

**Table S3: Utilities scores associated with model health states**

| In untreated and uncured patients | |  |
| --- | --- | --- |
|  | F0-F3 | 0.74 |
|  | CC | 0.71 |
|  | DC | 0.66 |
|  | HCC | 0.66 |
| During Treatment | |  |
|  | F0-F3 | 0.78 |
|  | CC | 0.75 |
| In cured patients | |  |
|  | F0-F3 | 0.81 |
|  | CC | 0.77 |
|  | DC | 0.72 |
|  | HCC | 0.66 |

Second, given the limited data on SF-6D utility scores for the HCC health state and the findings of two studies suggesting no or little differences in utility scores at the DC and HCC health states^12,13^, we made the conservative hypothesis that the HCC health state had the same utility score as the DC health state.

This choice is justified by the fact that few data have been reported in the literature on SF-6D utility values in patients with HCC, and the numbers of patients with HCC reported in the concerned studies were very small (<30). In Hsu et al.^12^, the mean SF-6D utility score estimated in a small group of patients with HCC (n=20) was similar to that obtained in a sample of 137 patients with CC or DC (mean values (SD): 0.61 (0.12) and 0.61 (0.12), respectively). In another study by Chong et al.^13^, the utility scores measured in a small (n=15) group of patients with HCC using the Visual Analogic Scale and the EuroQol Index^14^ were barely equal to those of patients with DC (n=9). However, using the Health Utilities Index Mark 3^15^, the utility scores differed slightly between the two groups of patients (with DC *versus* with HCC). Given the low numbers of patients in both studies and the absence of alternative sources of information on the utility scores in patients with HCC, we chose to assume that the utility score for patients in the HCC health state was the same as that for patients in the DC health state. This assumption is rather conservative, as the number of life years (LY) spent in the HCC health state was higher in the untreated simulated cohorts than in the treated cohort (and consequently, the number of QALYs attributed to this health state is overestimated in both cohorts, but more in the untreated cohort than in the treated cohort). In addition, the number of LY spent in the HCC health state is limited (as only a small number of patients will develop HCC) and HCC occurred late in life.

- 1. ***Transition probabilities***

In the absence of SSA country-specific data, transition probabilities between fibrosis stages without treatment were obtained from a meta-analysis^16^, while probabilities of transition from F3 to advanced stages (CC, DC and HCC) were derived from Dienstag et al. (2011)^17^.

Transition probabilities (according to viremic status) from CC (to DC and to HCC) were derived from Nahon et al. (2018)^18^. This study used data from a cohort of 1270 HCV positive patients with compensated cirrhosis enrolled from 2006 to 2012 in 35 hospitals in France. Patients were classified as follows: i) receiving DAAs treatment (DAAs group, n=336), ii) achieving sustained virologic response following an Interferon-based regimen (SVR-IFN group, n=495), iii) never receiving DAAs treatment and never achieving SVR after an Interferon-based regimen (non-SVR group, n=439).

The transition probabilities were calculated using the incidence rate^19^ over 3 years obtained from the data of the survival curves reported in the paper:

$$3 years probabilities= \frac{Number of events over the 3-year period}{Number of \text{Person-Year} over the 3- year period}$$

As the number of persons at risk is given annually, the exact numbers of Person-Years over the 3-year period were approximated assuming that changes in the number of persons at risk occurred in the middle of the year. So if over 3 years, 100 persons were at risk in the first two years and 75 in the third, the total number of Person-Years was equal to: $\left( 100+75+75 \right)\times1+\left( 100-75 \right)\times0.5=262.5$.

Annual probabilities of death associated with CC and DC were obtained from Dienstag et al. (2011)^17^ and Planas et al. (2004)^20^, respectively.

As therapeutic options for patients with HCC living in the three study countries Cameroon, Côte d’Ivoire and Senegal were very limited, we estimated the annual probability of death using HCC survival data in patients who did not receive specific treatment for liver cancer in a multi-country observational study conducted in SSA^21^.

All-cause mortality probabilities according to age and country were derived from WHO mortality tables^22^.

The probability of reinfection or relapse after treatment in patients achieving SVR was obtained from a meta-analysis including studies conducted in mono-HCV infected patients living in high-income countries and without specific risk factors for re-infection^23^. In the deterministic sensitivity analysis, we also considered a cohort at high risk of yearly reinfection (0.032 *versus* 0.002) using the probabilities of re-infection estimated in a group of HIV/HCV coinfected patients obtained from the same meta-analysis.

*Conversion of transition probabilities*

In the first cycle, yearly probabilities of transition ($p_{i}$) were converted to 24-week probabilities ($p_{j})$ using the following formula: $p_{j}=1-\left( 1-p_{i} \right)^{\frac{d_{j}}{d_{i}}}$ where $d_{i}$and $d_{j}$are the time horizons of each probability in the same unit (here 52 and 24 weeks).

WHO non-CHC related mortality probabilities^24^ and 3-year probabilities derived from Nahon (2018)^25^ are also reported on a longer time horizon than one year (5 and 3 years, respectively). They were converted in yearly probabilities using the same formula.

- 1. ***Costs***

Data on the quantities and unit cost used in the analyses are summarized in Table S4 (pages 16-17) and additional information is provided below.

1. *Quantities of healthcare resources used*

Data on annual use of healthcare resources for patients at the different stages of CHC were obtained from the TAC pilot trial for the treatment period and interviews with experts on hepatitis in the study countries in accordance with 2018 WHO guidelines^26^. Experts were asked questions about the number of visits to practitioners, hospitalizations, laboratory tests, and drugs, in the absence of HCV treatment and according to i) different stages of fibrosis, and ii) the presence or absence of compensated/decompensated cirrhosis or HCC.

In particular, the following assumptions were made:

- the proportions of patients seeking care in hospitals because of liver complications at the CC, DC and HCC health states were set at 20% based on expert opinions (this assumption is conservative as it underestimated the costs in the untreated cohorts).
  - the hospitalization duration was set at 14.3 days per year in the CC health state, 21.45 days per year in the DC health state, and 21.45 days per year in the HCC health state.
- the proportions of patients with ascites and gastroesophageal bleeding in the DC health state were set at 78.4% and 31.0%, respectively, based on Nahon et al. (2018)^25^.
  - for each ascites episode (i.e., 4 per year) or gastrointestinal bleeding episode (i.e., 1 per year), patients who sought care (i.e., 20% of all patients) were hospitalized: we assumed that i) they had one day of hospitalization per ascites episode (for ascites punctures) and 5 days of hospitalization per episode of gastrointestinal bleeding; ii) they received antibiotics (either 2000 mg/day of Amoxicillin or 1500mg/day of Ciprofloxacin or 1500mg/day of Metronidazole for a median of 8.5 days (between 7 and 10 days);
- at the most advanced stages of the disease (DC and HCC), patients who sought care (i.e. 20%) received analgesic drugs. As opioid-based drugs such as morphine were not widely available in the study countries at the time of our study, we considered that patients at DC and HCC stages received 6000 mg/day of Paracetamol and 180 mg/day of Codeine, corresponding to the maximum daily recommended doses for these drugs. No other treatment or care (e.g., chemotherapy, surgery for liver tumor resection or liver transplantation) was considered at the DC and HCC stages as these medical acts are very rare (liver tumor resection) or even unavailable (chemotherapy and liver transplantation) in the study countries.

1. *Unit cost of healthcare resources*

The respective unit costs of healthcare resources (except DAAs) were obtained for the year 2017 using data collection in healthcare facilities, reference laboratories and national drugs procurement agencies in the three study countries.

- Medical consultations, biological and non-biological tests

The unit cost of medical consultations, biological and non-biological tests, were collected in the following sites (including the four study sites and the main reference laboratories in each country), respectively in Cameroon, Côte d’Ivoire and Senegal:

- Clinique Cathédrale in Yaounde (study site), Centre Pasteur du Cameroun (reference laboratory), Centre de recherches sur les maladies émergentes, re-émergentes et la médecine nucléaire (reference laboratory for HIV and viral hepatitis in Yaoundé), Central Hospital of Yaounde (study site).
- University Hospital Medical Center at Treichville in Abidjan and University Hospital Medical Center at Yopougon in Abidjan.
- Centre médical Interarmées (CMIA) in Dakar (hospital), Centre hospitalier national universitaire (CHNU) de Fann in Dakar (study site) and Dakar Pasteur Institute (reference laboratory).

Unit costs were selected based on the following approach: i) prioritizing non-profit or public healthcare facilities and laboratories to obtain unit prices which provided the best approximation of the actual cost (and thus of the opportunity cost); ii) excluding healthcare facilities and laboratories whose prices were subsidized or whose access was restricted to specific populations (e.g., soldiers). When several unit costs were available for the same medical resource in a study country, we selected the lowest one.

In Cameroon, unit prices used in the analysis primarily came from the Central Hospital of Yaounde (and for a minority of resources from the Centre Pasteur du Cameroun). In Côte d’Ivoire, most unit prices were obtained from the University Hospital Medical Center at Yopougon in Abidjan. In Senegal, unit prices primarily came from the Centre Hospitalier National Universitaire (CHNU) de Fann in Dakar and some from the Dakar Pasteur Institute.

- Drugs (other than DAAs)

Unit prices for all drugs other than DAAs were obtained from the price lists of the national drugs procurement agency of each study country. When several prices were available for different galenic formulations and doses, we used the price of the formulation most commonly used.

- Hospitalization

One-night hospitalization prices were extracted for each country from the WHO-CHOICE database which is an international health service delivery cost database^27^.

- DAAs

In the first scenario (S1) we considered DAAs prices of originator pharmaceutical companies observed during the study period (2016-2017). These were valued using the WHO Global Price Reporting Mechanism database when their prices were available for the study countries (i.e., for sofosbuvir+ribavirin and the fixed-dose combination sofobuvir/ledipasvir)^28^ and if not, using prices reported by Médecins Sans Frontières (for sofosbuvir+daclatasvir and the fixed-dose combination sofosbuvir/velpatasvir)^29^.

In the second scenario (S2), we considered DAAs prices of generic manufacturers using data from WHO when their prices were available in the study countries (i.e. for sofosbuvir/ribavirin and sofobuvir/ledipasvir available as fixed-dose combinations)^30^ and if not, using prices reported by Médecins Sans Frontières (for sofosbuvir/daclatasvir and sofosbuvir/velpatasvir, both as fixe-dose combinations)^29^.

**Table S4. Unit costs per country (US dollars, 2017) and annual quantities of healthcare resources associated with model health states**

|  | **Unit costs per country** | | | **Quantity of healthcare resources used per health state** | | | |
| --- | --- | --- | --- | --- | --- | --- | --- |
|  | Cameroon | Côte d’Ivoire | Senegal | Treatment | Compensated Cirrhosis | Decompensated Cirrhosis | Hepatocellular Carcinoma |
| **Biological and non-biological tests** | | | | | | | |
| Albumin | 6.2 | 1.7 | 8.6 | 2* | 2 | 2 | 0 |
| HCV RNA PCR | 107.4 | 48.1 | 77.3 | 2^¤^ | 0 | 0 | 0 |
| HCV genotyping | 165 | 137.5 | 151.2 | 0 or 1^µ^ | 0 | 0 | 0 |
| Total bilirubin | 3.4 | 1.7 | 5.2 | 2^#^ | 2 | 2 | 0 |
| Creatinine | 2.2 | 1.7 | 3.4 | 2^#^ | 2 | 2 | 0 |
| Liver Ultrasound | 13.7 | 17.2 | 25.8 | 2* | 2 | 2 | 0 |
| EGD | 61.9 | 61.9 | 61.9 | 2* | 0.5^+^ | 0.5 | 0.5 |
| GGT | 3.6 | 5.2 | 5.2 | 2* | 2 | 2 | 0 |
| Prothrombin Rate | 5.2 | 8.6 | 8.6 | 2* | 2 | 2 | 0 |
| Glycemia | 2.1 | 1.7 | 4.3 | 0 | 0 | 0 | 0 |
| Platelet count | 6.2 | 5.2 | 6.9 | 3^#^ | 2 | 2 | 0 |
| Immunology | 14.6 | 20.6 | 17.6 | 0 | 0 | 0 | 0 |
| Ionogramme (K+, Na+, Cl-) | 7.9 | 5.2 | 17.2 | 0 | 0 | 0 | 0 |
| Ascites drain | 2.4 | 2.3 | 2.2 | 0 | 0 | 3.1 | 0 |
| Chest Radiography | 11.5 | 8.6 | 17.2 | 1 | 0 | 0 | 0 |
| HBsAg RDT | 12 | 10.3 | 3.4 | 1 | 0 | 0 | 0 |
| HCV Ab RDT | 12 | 10.3 | 3.4 | 1 | 0 | 0 | 0 |
| HIV Ab | 12 | 12 | 12 | 1 | 0 | 0 | 0 |
| Pregnancy test | 2.6 | 2.6 | 2.6 | 1^$^ | 0 | 0 | 0 |
| Transaminases | 8.6 | 8.6 | 8.6 | 2^#^ | 0 | 0 | 0 |
| **Medical consultations** | | | | | | | |
| Infectiologist | 4.5 | 6 | 8.6 | 3^#^ | 2 | 2 | 2 |
| Hepatologist | 6 | 4.5 | 8.6 |  |  |  |  |
| Internist | 4.5 | 6 | 8.6 |  |  |  |  |
| Nurse counselling | 1.7 | 1.7 | 1.7 | 1 | 0 | 0 | 0 |
| **Hospitalization** | | | | | | | |
| Inpatient stay | 2.4 | 2.3 | 2.2 | 0 | 2.86 | 4.6 | 4.29 |

| **Table S4 (continued)** | | | | | | | | |
| --- | --- | --- | --- | --- | --- | --- | --- | --- |
|  | | **Unit costs per country** | | | **Quantity of healthcare resources used per health state** | | | |
|  | | Cameroon | Côte d’Ivoire | Senegal | Treatment | Compensated Cirrhosis | Decompensated Cirrhosis | Hepatocellular Carcinoma |
| **Drugs (except DAA)** | | | | | | | | |
| ***Analgesic*** | |  |  |  | 0 | 0 | 0 | 0 |
|  | Paracetamol 500mg | 0.1 | 0.1 | 0.1 | 0 | 0 | 0 | 364.25 |
|  | Codeine 30mg | 0.3 | 0.3 | 0.3 | 0 | 0 | 0 | 364.25 |
| ***Antibiotics*** | |  |  |  | 0 | 0 | 0 | 0 |
|  | Amoxicillin 500mg | 0.2 | 0.2 | 0.2 | 0 | 0 | 5.9 | 2.83 |
|  | Ciprofloxacin 500mg | 0.3 | 0.2 | 0.1 | 0 | 0 | 5.9 | 2.83 |
|  | Metronidazole 5mg/ml | 0.1 | 0.1 | 0.1 | 0 | 0 | 5.9 | 2.83 |

^#^ One additional test was performed for patients treated with ribavirin (RBV).

* Only for patient with compensated cirrhosis.

^µ^ HCV genotyping was only performed for patients receiving genotype specific regimens (i.e. sofosbuvir/ribavirin and sofosbuvir/velpatasvir).

^$^ Only for women of childbearing age.

Abbreviations: EGD: Esophagogastroduodenoscopy; FBC: Full Blood Count; GGT: Gamma-glutamyl transferase; HBsAg RDT: Surface antigen of the hepatitis B virus Rapid Diagnostic Test; HCV Ab RDT =Hepatitis C Antibody Rapid Diagnostic Test; HCV RNA PCR = Polymerase chain reaction (PCR) of Hepatitis C virus’ (HCV) Ribonucleic acid (RNA); HIV Ab: Human Immunodeficiency Viruses Antibody.

1. *Computation of the model’s health state costs*

The total cost of each model’s health state was then computed for each country as the sum of each healthcare resource used to care for patients in a given health state, multiplied by its respective unit cost. Costs estimations for each model’s health state are provided in the Supplemental Table S5 p.19-20. For example:

- The total cost of a patient in the health state “Treatment at F0-F3 stage using originator sofosbuvir/ledispavir” is estimated at **US$1692.6 in Cameroon** computed as the sum of the cost of originator SOF/LDV ($1201.2) + the costs of laboratory tests corresponding to genotype-dependent regimen ($474.7) + the costs of consultations ($16.7).
- The total cost of a patient in the health state “Treatment at F0-F3 stage using originator sofosbuvir/ribavirin” is estimated at **US$1660.1 in Cameroon** computed as the sum of the cost of originator SOF/RBV ($1035.9) + the cost of laboratory tests corresponding to genotype-dependent regimen ($474.7) + consultations ($16.7) + additional tests ($127.8) + additional consultation ($5).

**Table S5: Model’s health state costs (in US dollars, 2017)**

|  |  |  |  | Cameroon | Côte d’Ivoire | Senegal |
| --- | --- | --- | --- | --- | --- | --- |
| Treatment while at mild fibrosis (F0-F3) stage | Tests* (1) | |  |  |  |  |
|  |  | With genotype-dependent regimen | | 474.7 | 314.8 | 397.3 |
|  |  | With pangenotypic regimen | | 309.7 | 177.3 | 246.1 |
|  | Consultations* (2) | |  | 16.7 | 18.2 | 27.5 |
|  | Drugs (3) | |  |  |  |  |
|  |  | SOF/RBV | Originator | 1035.9 | 1035.9 | 1035.9 |
|  |  |  | Generic | 405.6 | 405.6 | 405.6 |
|  |  | SOF/LDV | Originator | 1201.2 | 1201.2 | 1201.2 |
|  |  |  | Generic | 429.0 | 429.0 | 429.0 |
|  |  | SOF/DCV | Originator | 1251.0 | 1251.0 | 1251.0 |
|  |  |  | Generic | 195.0 | 195.0 | 195.0 |
|  |  | SOF/VEL | Originator | 900.0 | 900.0 | 900.0 |
|  |  |  | Generic | 450.0 | 450.0 | 450.0 |
|  | Total (1+2+3) | |  |  |  |  |
|  |  | SOF/RBV | Originator | 1660.1 | 1439.7 | 1570.7 |
|  |  |  | Generic | 1029.8 | 809.4 | 940.4 |
|  |  | SOF/LDV | Originator | 1692.6 | 1534.2 | 1626.0 |
|  |  |  | Generic | 920.4 | 762.0 | 853.8 |
|  |  | SOF/DCV | Originator | 1577.4 | 1446.5 | 1524.6 |
|  |  |  | Generic | 521.4 | 390.5 | 468.6 |
|  |  | SOF/VEL | Originator | 1226.4 | 1095.5 | 1173.6 |
|  |  |  | Generic | 776.4 | 645.5 | 723.6 |
| Treatment while at Compensated Cirrhosis (CC) stage | Tests* | |  |  |  |  |
|  |  | With genotype-dependent regimen | | 655.9 | 503.9 | 617.3 |
|  |  | With pangenotypic regimen | | 490.9 | 366.4 | 466.0 |
|  | Consultations* | |  | 16.7 | 18.2 | 27.5 |
|  | Drugs | | Originator | *DAA costs are identical to those of patients treated at mild fibrosis stage* | | |
|  |  |  | Generic |  |  |  |
|  | Total | |  |  |  |  |
|  |  | SOF/RBV | Originator | 1841.3 | 1628.8 | 1790.7 |
|  |  |  | Generic | 1211.0 | 998.5 | 1160.4 |
|  |  | SOF/LDV | Originator | 1873.8 | 1723.3 | 1846.0 |
|  |  |  | Generic | 1101.6 | 951.1 | 1073.8 |
|  |  | SOF/DCV | Originator | 1758.6 | 1635.6 | 1744.5 |
|  |  |  | Generic | 702.6 | 579.6 | 688.5 |
|  |  | SOF/VEL | Originator | 1407.6 | 1284.6 | 1393.5 |
|  |  |  | Generic | 957.6 | 834.6 | 943.5 |
| **Table S5 (continued)** | | | | | | |
|  |  |  |  | Cameroon | Côte d’Ivoire | Senegal |
| Mild Fibrosis (F0-F3) | Tests | |  | 0 | 0 | 0 |
|  | Consultations | |  | 0 | 0 | 0 |
|  | Drugs | |  | 0 | 0 | 0 |
|  | Total | |  | 0 | 0 | 0 |
| Compensated Cirrhosis (CC) | Tests | |  | 112.0 | 113.4 | 158.1 |
|  | Consultations | |  | 10.0 | 11.0 | 17.2 |
|  | Drugs | |  | 0.0 | 0.0 | 0.0 |
|  | Hospitalisation | |  | 6.8 | 6.5 | 6.2 |
|  | Total | |  | 128.7 | 130.9 | 181.5 |
| Decompensated Cirrhosis (DC) | Tests | |  | 119.4 | 120.5 | 164.9 |
|  | Consultations | |  | 10.0 | 11.0 | 17.2 |
|  | Drugs | |  | 2.9 | 2.5 | 2.0 |
|  | Hospitalisation | |  | 10.9 | 10.4 | 9.9 |
|  | Total | |  | 143.2 | 144.4 | 194.0 |
| Hepatocellular Carcinoma (HCC) | Tests | |  | 30.9 | 30.9 | 30.9 |
|  | Consultations | |  | 10.0 | 11.0 | 17.2 |
|  | Drugs | |  | 131.5 | 136.8 | 142.0 |
|  | Hospitalisation | |  | 10.1 | 9.7 | 9.2 |
|  | Total | |  | 182.6 | 188.4 | 199.3 |

** One additional visit is required when RBV is prescribed, corresponding to a supplementary cost of USD 127.80, 65.30, 101.40 for tests, and USD 5.00, 5.50, 8.60 for the consultation, in Cameroon, Côte d’Ivoire and Senegal, respectively.*

Abbreviations: CC: Compensated Cirrhosis; DC: Decompensated Cirrhosis; F0, F1, F2, F3: METAVIR fibrosis stages; HCC: Hepatocellular Carcinoma; SOF/DCV: Sofosbuvir+Daclatasvir; SOF/LDV: Sofosbuvir+Ledipasvir; SOF/RBV: Sofosbuvir/Ribavirin; SOF/VEL: Sofosbuvir+Velpatasvir.

1. **Probabilistic Sensitivity Analysis (PSA)**

The PSA was conducted using a Monte Carlo simulation with 10,000 iterations. In this analysis, all parameters varied simultaneously, with multiple sets of parameter values being sampled from a priori–defined probability distributions. Based on standard practice^31^, transition probabilities and utility scores estimates were assumed to follow a Beta distribution and costs were assumed to follow a Gamma distribution. When studies did not report the 95% CI associated to transition probabilities, these latter were computed using the Wilson score formula^32^. Distribution parameters were then derived using a Newton type optimization method to match the 2.5^th^ and 97.5^th^ percentiles of the confidence intervals. The distribution of the SVR rates and the corresponding 95% CI were estimated using data from the TAC pilot trial with the standard bootstrap method (for each of the 10,000 iterations of the PSA^33^, a sample of 120 patients with replacement was drawn and the mean SVR rate was calculated in the new sample obtained; the 95% CI was then obtained using the percentile approach). The same procedure was used for the utility scores. Gamma distributions were parametrized for each health state cost using the method of moments^34^ applied to the empirical mean μ and empirical standard deviation σ. A function with one outer loop was developed for each country.

- 1. ***Calculating confidence intervals of transition probabilities***

When the confidence intervals (CI) of transition probabilities were not reported by a study, they were calculated using the Wilson score formula^32^:

$CI\left( p \right)_{\alpha}=\left[ \frac{p+\left( \frac{z^{2}}{2*n} \right)}{1+\left( \frac{z^{2}}{n} \right)}\pm\frac{z}{1+\left( \frac{z^{2}}{n} \right)}\times\sqrt{\frac{p\times\left( 1-p \right)}{n}+\frac{z^{2}}{4\times n^{2}}} \right]$
where $p$ is the probability, $z$ is the $1-\frac{\alpha}{2}$ [quantile](https://en.wikipedia.org/wiki/Quantile) of a [standard normal distribution](https://en.wikipedia.org/wiki/Standard_normal_distribution) and $\alpha$ the significance level. The Wilson score interval was preferred over the normal approximation interval because the sample size and the probability were both small, so the normal approximation was not indicated^35^.

- 1. ***Parametrizing Beta distribution in the PSA***

Given two points $\left( x_{i}, p_{i} \right), i=1, 2$, where $p_{i}$ is a probability on a scaled, recentered beta cumulative distribution function (CDF), and $x_{i}$ is a value on the same CDF, we solved the following equation to obtain the $\alpha$ and$\beta$parameters of the beta distribution:

$F\left( \alpha,\beta;x_{i} \right)= p_{i}, i=1,2$ where $F\left( \alpha,\beta;x_{i} \right)= \frac{\Gamma\left( \alpha+\beta\right)}{\Gamma\left( \alpha\right)\Gamma\left( \beta\right)}\int_{0}^{x} t^{\alpha-1}\left( 1-t \right)^{\beta-1}dt$

where $x_{1}$ and $x_{2}$ corresponded to the lower and upper bounds of the confidence intervals either reported in the literature or calculated, and $p_{1}$and $p_{2}$ were equal to 0.025 and 0.975. As there was no closed-form general solution of the two-equation system, we used a numerical method to solve it. To avoid a unreliable solution of the optimization problem due to near zero and near one values of $p_{i}$, we minimised the sum of squared differences of the logit of $p_{i}$ and the logit of the CDF. We performed Schnabel’s (1985)^36^ *nlm* minimisation method implemented in R^37^.

We applied this method to parametrize all beta distributions.

- 1. ***Non-parametric bootstrap for SVR rates and for utilities measured in the TAC pilot trial***

To compute the bootstrap distributions of SVR rates and utilities, we used data from the TAC pilot trial. At each of the 10,000 iterations of the PSA, a sample of 120 patients with replacement was drawn and the mean SVR rate calculated in the new sample obtained. The 95% CI was then obtained using the 2.5^th^ and 97.5^th^ percentiles of the distribution. The same procedure was used for the utility scores.

- 1. ***Parametrizing cost distribution in the PSA***

1. *Introducing uncertainty in costs*

For each health state and each country, the total cost was computed as the product of unit prices and the quantities of healthcare resources used. As unit prices were measured on site and quantities were estimated by hepatitis experts, no uncertainty was initially present in the costs at each health state, and consequently the costs were deterministic.

There are very few data in the literature on the costs related to HCV and liver complications^38–41^, and at the time of the study, no costs data were available for the African continent. In McAdam (2011)^41^, the dispersion of per-patient annual costs is reported for the CC, DC and HCC health states. In that study, all-cause health care services were collected using a retrospective analysis of administrative healthcare out-of-pocket reimbursement data in a large commercial insurer database from July 1, 2001, through June 30, 2010.

Given the absence of data in the sub-Saharan setting, we assumed that the coefficients of variation of costs for the CC, DC and HCC health states in our study were similar to those reported in McAdam (2011). Considering i) the total cost obtained for each health state and country in our study as the mean cost and ii) the coefficient of variation reported in McAdam (2011), we calculated the variance associated with the mean cost.

For example, we estimated the total cost of one year spent in the Compensated Cirrhosis (CC) health state at US$181.50 in Senegal. In McAdam (2011), the mean cost for patients in this health state was US$16,911 and the standard deviation was 659. The corresponding coefficient of variation is thus 0.039. Applying this coefficient of variation to the total costs estimated in our study (US$181.50) gave us a standard deviation of 7.07 (i.e. $181.5 \times\frac{659}{16911}$). Mean costs (SD) reported in McAdam (2011) for DC and HCC health states were US$41,943 (1129) and US$58,208 (2912), respectively.

1. *Parametrizing Gamma distributions for costs*

Given a mean cost $\mu$ and a standard deviation $\sigma$, we were then able to parametrize a gamma distribution for the cost at each disease stage using the moment method.

The Gamma distribution with parameters *shape* = a and *scale* = s has the following density function:

$f\left( x \right)=\frac{1}{s^{a} \Gamma\left( a \right)}x^{a-1} e^{-\left( \frac{x}{s} \right)}$ for x ≥ 0, a > 0 and s > 0. Where $\Gamma(x) = \int_{0}^{\infty} t^{x-1}exp\left( -t \right) dt$

The mean and variance of this distribution are $E(X) = a\times s$and $Var(X) = a\times s^{2}$.

Applying the moment method, we solved $\left\{ \begin{aligned} E\left( X \right)=\mu= a \times s \\ \mathrm{Var}\left( X \right)= \sigma^{2}= a\times s^{2} \end{aligned} \right.$ where$\mu$ is the empirical mean and $\sigma^{2}$ the empirical variance.

Thus $a= \frac{\mu^{2}}{\sigma^{2}}$ and $s= \frac{\sigma^{2}}{\mu}$.

With the example of CC in Senegal, the method gave us:

$a= \frac{{181.5}^{2}}{{7.1}^{2}}=$ 658.5 and $s= \frac{{7.1}^{2}}{181.5}=0.28.$

Therefore, the distribution of the cost for the CC disease stage for Senegal was *Gamma(658.5; 0.28)*.

1. **Model validity**

We tested the internal validity of our model i) by simulating the treated fictive cohorts with an SVR rate equal to zero (all results should be identical for the treated and untreated cohorts); ii) by assuming that the utilities and then the costs associated with health states were all equal to zero (the expected lifetime QALYs and costs, respectively should be equal to zero); iii) by assuming that all treatment strategies have the same SVR rate as that for the sofosbuvir/daclatasvir regimen, all other things being equal (all treatment strategies should therefore have the same expected ICERs).

*Internal consistency of the model*

When we simulated the treated cohorts with an SVR rate equal to zero, results for the treated and untreated cohorts were identical. In the second extreme value analysis (where utilities and then costs associated with health states were equal to zero), expected lifetime QALYs and costs were equal to zero for all treatment strategies. In the last scenario, where all treatment strategies are assumed to have the same SVR rate as that of the daclatasvir-based regimen, all other things being equal, all treatment strategies had the same expected ICERs in both scenarios.

**Table S6. Model parameter contributions to the variability of cost-effectiveness results**

| **Parameter** | **R-squared** | **IQR** | **min** | **max** | **95% CI** |
| --- | --- | --- | --- | --- | --- |
| U(CC) in untreated or uncured patients | 31.762 | 0.710 [0.702-0.718] | 0.666 | 0.757 | [0.686-0.734] |
| U(DC or HCC) in untreated or uncured patients | 31.762 | 0.662 [0.654-0.669] | 0.62 | 0.706 | [0.640-0.684] |
| U(F0-F3) in untreated or uncured patients | 31.762 | 0.743 [0.734-0.751] | 0.696 | 0.792 | [0.718-0.768] |
| U(CC) in cured patients | 25.084 | 0.769 [0.762-0.776] | 0.727 | 0.803 | [0.749-0.789] |
| U(DC) in cured patients | 25.084 | 0.717 [0.711-0.724] | 0.678 | 0.749 | [0.698-0.736] |
| U(F0-F3) in cured patients | 25.084 | 0.805 [0.798-0.812] | 0.76 | 0.841 | [0.783-0.826] |
| F3 → CC in untreated or uncured patients | 13.833 | 0.081 [0.068-0.095] | 0.015 | 0.181 | [0.047-0.126] |
| F2 → F3 in untreated or uncured patients | 7.288 | 0.111 [0.098-0.125] | 0.052 | 0.205 | [0.077-0.152] |
| SVR12 in treated patients | 5.824 | 0.935 [0.918-0.953] | 0.813 | 1.000 | [0.874-0.988] |
| F3 → CHC-related mortality | 3.686 | 0.009 [0.006-0.012] | 0.001 | 0.034 | [0.003-0.019] |
| F3 → HCC in untreated or uncured patients | 3.347 | 0.012 [0.009-0.015] | 0.002 | 0.036 | [0.005-0.023] |
| F3 → DC in untreated or uncured patients | 1.481 | 0.012 [0.009-0.015] | 0.002 | 0.041 | [0.005-0.023] |
| CC → CHC-related mortality | 1.389 | 0.027 [0.022-0.032] | 0.007 | 0.068 | [0.014-0.045] |
| F1 → F2 in untreated or uncured patients | 0.651 | 0.059 [0.055-0.063] | 0.039 | 0.085 | [0.048-0.072] |
| C(treatment) | 0.499 | 1524.500 [1511.509-1537.880] | 1443.601 | 1606.309 | [1486.731-1564.518] |
| CC → CHC-related mortality | 0.483 | 0.027 [0.022-0.032] | 0.006 | 0.068 | [0.014-0.045] |
| U(CC) on treatment | 0.400 | 0.750 [0.743-0.757] | 0.71 | 0.789 | [0.729-0.770] |
| U(F0-F3) on treatment | 0.400 | 0.785 [0.778-0.792] | 0.742 | 0.825 | [0.763-0.806] |
| C(CC) in untreated or uncured patients | 0.254 | 181.422 [176.796-186.261] | 155.046 | 207.241 | [168.127-195.642] |
| C(CC) in cured patients | 0.254 | 181.422 [176.796-186.261] | 155.046 | 207.241 | [168.127-195.642] |
| CC → HCC in untreated or uncured patients | 0.144 | 0.042 [0.039-0.045] | 0.028 | 0.059 | [0.034-0.051] |
| DC → CHC-related mortality | 0.113 | 0.133 [0.124-0.143] | 0.087 | 0.197 | [0.107-0.163] |
| Annual reinfection probability | 0.074 | 0.002 [0.001-0.002] | 0 | 0.004 | [0.001-0.003] |
| DC → HCC in untreated or uncured patients | 0.046 | 0.068 [0.061-0.076] | 0.034 | 0.119 | [0.049-0.091] |
| CC → HCC in cured patients | 0.039 | 0.015 [0.012-0.019] | 0.003 | 0.043 | [0.007-0.029] |
| CC → DC in untreated or uncured patients | 0.022 | 0.042 [0.039-0.044] | 0.028 | 0.058 | [0.034-0.050] |

| **Table S6 (continued)** | | | | | |
| --- | --- | --- | --- | --- | --- |
| **Parameter** | **R-squared** | **IQR** | **min** | **max** | **95% CI** |
| CC → DC in cured patients | 0.022 | 0.025 [0.021-0.029] | 0.007 | 0.06 | [0.014-0.040] |
| C(DC) in in untreated or uncured patients | 0.014 | 194.063 [190.494-197.412] | 173.912 | 216.22 | [183.674-204.091] |
| C(HCC) | 0.012 | 199.170 [192.534-206.139] | 163.712 | 246.214 | [179.833-220.008] |
| HCC → CHC-related mortality | 0.009 | 0.905 [0.891-0.918] | 0.815 | 0.963 | [0.860-0.941] |
| DC → CHC-related mortality | 0.002 | 0.133 [0.124-0.143] | 0.08 | 0.193 | [0.107-0.162] |
| DC → HCC in cured patients | 0.001 | 0.068 [0.061-0.075] | 0.036 | 0.119 | [0.049-0.091] |
| C(DC) in cured patients | 0.000 | 193.914 [190.510-197.439] | 176.207 | 213.984 | [183.996-204.183] |
| F0 → F1 in untreated or uncured patients | 0.000 | 0.081 [0.070-0.093] | 0.028 | 0.162 | [0.052-0.119] |

Abbreviations: C: Costs; CC: Compensated Cirrhosis; CHC: Chronic Hepatitis C; DC: Decompensated Cirrhosis; F0, F1, F2, F3: METAVIR fibrosis stages; HCC: Hepatocellular Carcinoma; SVR12: Sustained Virologic Response measured at week 12 after treatment end; U: Utility.

**Figure S2: Deterministic sensitivity analysis for Scenario 1 (originator prices) (Fig. S2a) and Scenario 2 (generic prices) (Fig. S2b)**

**Figure S2a**


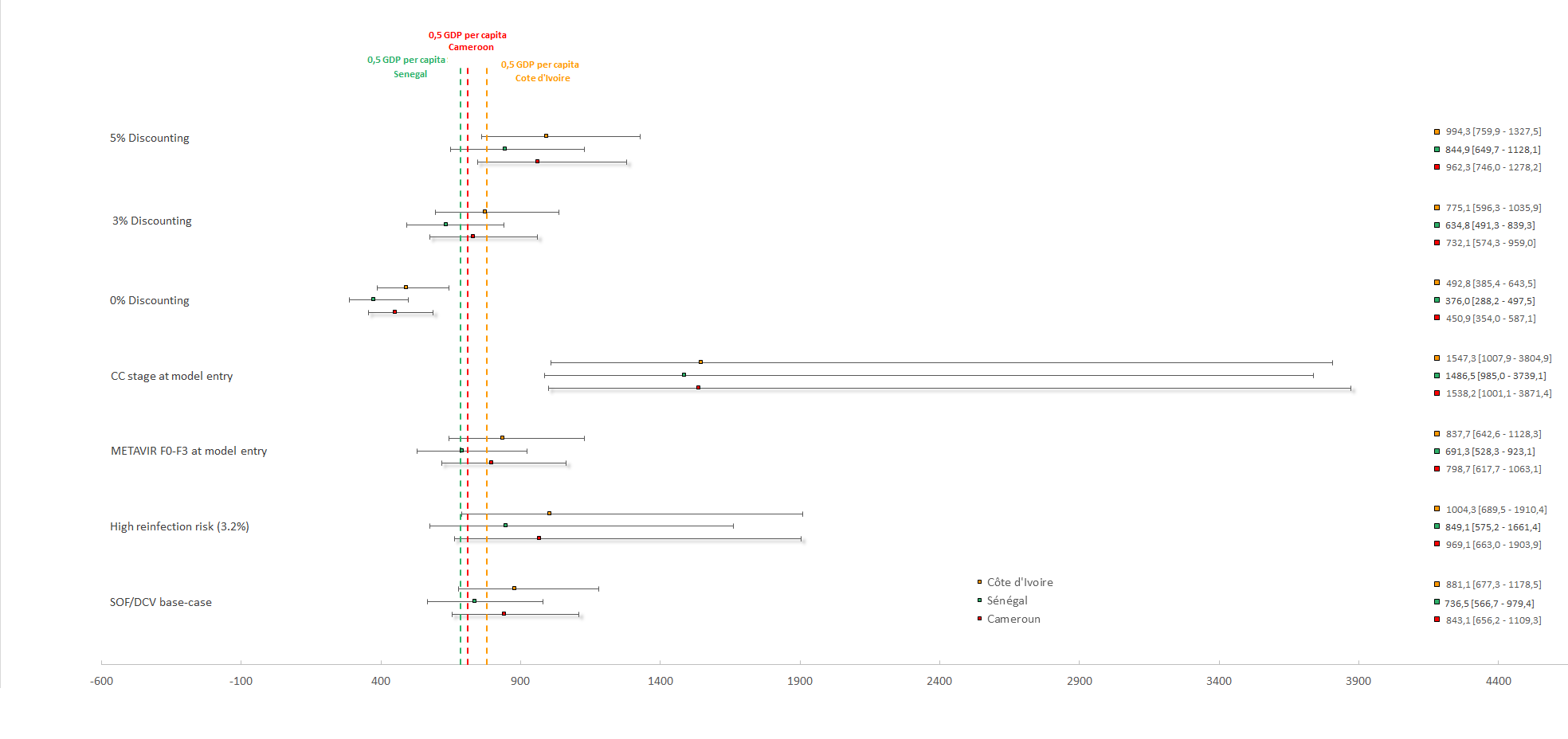


**Figure S2b**


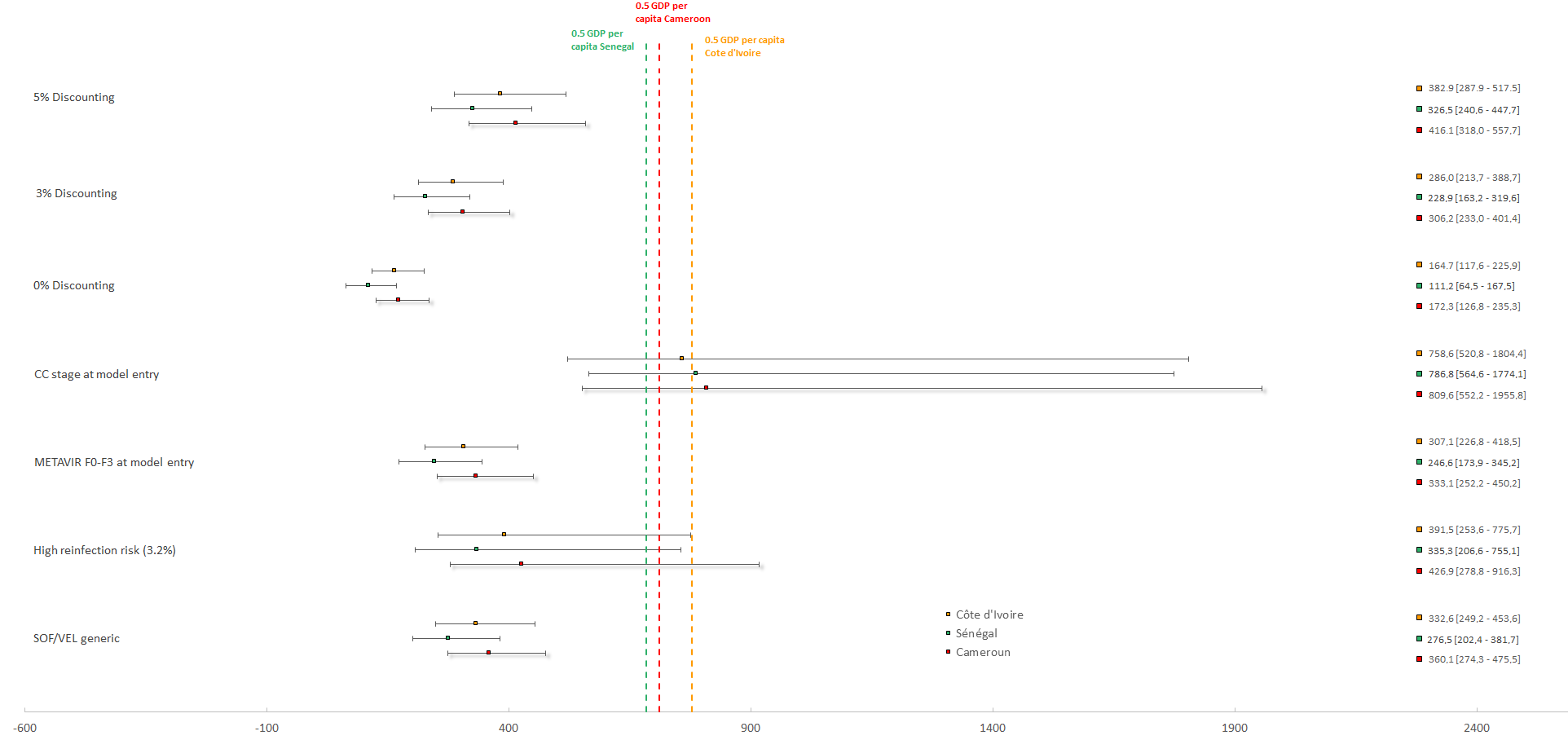


**Legend:** The horizontal axis shows the incremental cost-effectiveness ratios (in US dollars at 2017 prices) per quality-adjusted life-year for each scenario listed on the vertical axis. The colored squares indicate the mean expected ICERs, while the horizontal lines indicate the respective 95% CI around the mean expected values for each of the three study country (yellow for Côte d’Ivoire, green for Senegal, and red for Cameroon). The corresponding values are indicated on the right. The colored (green, red and yellow) vertical lines indicate the cost-effectiveness thresholds of 0.5 times the GDP per capita in 2017 for each of the three study countries (i.e., US$683.5 in Senegal, US$711 in Cameroon, US$778.5 in Côte d’Ivoire, respectively).

*Abbreviations: CI: Confidence Intervals; GDP: Gross Domestic Product; ICERs: incremental cost-effectiveness ratios.*

**References**

1. Pol S, Bourliere M, Lucier S, et al. Safety and efficacy of daclatasvir-sofosbuvir in HCV genotype 1-mono-infected patients. *J Hepatol*. 2017;66(1):39-47. doi:10.1016/j.jhep.2016.08.021

2. Carrat F, Fontaine H, Dorival C, et al. Clinical outcomes in patients with chronic hepatitis C after direct-acting antiviral treatment: a prospective cohort study. *Lancet Lond Engl*. 2019;393(10179):1453-1464. doi:10.1016/S0140-6736(18)32111-1

3. Lawitz E, Mangia A, Wyles D, et al. Sofosbuvir for Previously Untreated Chronic Hepatitis C Infection. http://dx.doi.org/10.1056/NEJMoa1214853. doi:10.1056/NEJMoa1214853

4. Jacobson IM, Gordon SC, Kowdley KV, et al. Sofosbuvir for Hepatitis C Genotype 2 or 3 in Patients without Treatment Options. http://dx.doi.org/10.1056/NEJMoa1214854. doi:10.1056/NEJMoa1214854

5. Zeuzem S, Dusheiko GM, Salupere R, et al. Sofosbuvir and Ribavirin in HCV Genotypes 2 and 3. http://dx.doi.org/10.1056/NEJMoa1316145. doi:10.1056/NEJMoa1316145

6. Afdhal N, Zeuzem S, Kwo P, et al. Ledipasvir and Sofosbuvir for Untreated HCV Genotype 1 Infection. *N Engl J Med*. 2014;370(20):1889-1898. doi:10.1056/NEJMoa1402454

7. Kowdley KV, Gordon SC, Reddy KR, et al. Ledipasvir and Sofosbuvir for 8 or 12 Weeks for Chronic HCV without Cirrhosis. *N Engl J Med*. 2014;370(20):1879-1888. doi:10.1056/NEJMoa1402355

8. Ware JE Jr, Kosinski M, Turner-Bowker DM, Sundaram M, Gandek B, Maruish ME. *User’s Manual for the SF-12v2 Health Survey Second Edition*. QualityMetric, Incorporated; 2009.

9. Sheffield U of. Measuring & Valuing Health - HEDS - Sections - ScHARR - The University of Sheffield. Accessed March 19, 2020. https://www.sheffield.ac.uk/scharr/sections/heds/mvh

10. Brazier JE, Roberts J. The estimation of a preference-based measure of health from the SF-12. *Med Care*. 2004;42(9):851-859. doi:10.1097/01.mlr.0000135827.18610.0d

11. Stepanova M, Younossi I, Racila A, Younossi ZM. Prediction of Health Utility Scores in Patients with Chronic Hepatitis C Using the Chronic Liver Disease Questionnaire-Hepatitis C Version (CLDQ-HCV). *Value Health J Int Soc Pharmacoeconomics Outcomes Res*. 2018;21(5):612-621. doi:10.1016/j.jval.2017.10.005

12. Hsu PC, Federico CA, Krajden M, et al. Health utilities and psychometric quality of life in patients with early- and late-stage hepatitis C virus infection. *J Gastroenterol Hepatol*. 2012;27(1):149-157. doi:10.1111/j.1440-1746.2011.06813.x

13. Chong CA, Gulamhussein A, Heathcote EJ, et al. Health-state utilities and quality of life in hepatitis C patients. *Am J Gastroenterol*. 2003;98(3):630-638. doi:10.1111/j.1572-0241.2003.07332.x

14. Dolan P. Modeling Valuations for EuroQol Health States. *Med Care*. 1997;35(11):1095–1108.

15. Spilker B. *Quality of Life and Pharmacoeconomics in Clinical Trials*. Lippincott-Raven; 1996.

16. Thein H-H, Yi Q, Dore GJ, Krahn MD. Estimation of stage-specific fibrosis progression rates in chronic hepatitis C virus infection: a meta-analysis and meta-regression. *Hepatol Baltim Md*. 2008;48(2):418-431. doi:10.1002/hep.22375

17. Dienstag JL, Ghany MG, Morgan TR, et al. A prospective study of the rate of progression in compensated, histologically advanced chronic hepatitis C. *Hepatol Baltim Md*. 2011;54(2):396-405. doi:10.1002/hep.24370

18. Nahon P, Layese R, Bourcier V, et al. Incidence of Hepatocellular Carcinoma After Direct Antiviral Therapy for HCV in Patients With Cirrhosis Included in Surveillance Programs. *Gastroenterology*. 2018;155(5):1436-1450.e6. doi:10.1053/j.gastro.2018.07.015

19. Last JM, ed. *A Dictionary of Public Health*. Oxford University Press; 2006.

20. Planas R, Ballesté B, Alvarez MA, et al. Natural history of decompensated hepatitis C virus-related cirrhosis. A study of 200 patients. *J Hepatol*. 2004;40(5):823-830. doi:10.1016/j.jhep.2004.01.005

21. Yang JD, Mohamed EA, Aziz AOA, et al. Characteristics, management, and outcomes of patients with hepatocellular carcinoma in Africa: a multicountry observational study from the Africa Liver Cancer Consortium. *Lancet Gastroenterol Hepatol*. 2017;2(2):103-111. doi:10.1016/S2468-1253(16)30161-3

22. WHO. Global Health Observatory data repository, life table by country. WHO. Published 2018. Accessed February 19, 2019. http://apps.who.int/gho/data/node.main.LIFECOUNTRY?lang=en

23. Simmons B, Saleem J, Hill A, Riley RD, Cooke GS. Risk of Late Relapse or Reinfection With Hepatitis C Virus After Achieving a Sustained Virological Response: A Systematic Review and Meta-analysis. *Clin Infect Dis Off Publ Infect Dis Soc Am*. 2016;62(6):683-694. doi:10.1093/cid/civ948

24. WHO. Global Health Observatory data repository, life table by country. WHO. Published 2018. Accessed February 19, 2019. http://apps.who.int/gho/data/node.main.LIFECOUNTRY?lang=en

25. Nahon P, Layese R, Bourcier V, et al. Incidence of Hepatocellular Carcinoma After Direct Antiviral Therapy for HCV in Patients With Cirrhosis Included in Surveillance Programs. *Gastroenterology*. 2018;155(5):1436-1450.e6. doi:10.1053/j.gastro.2018.07.015

26. WHO. WHO | Guidelines for the care and treatment of persons diagnosed with chronic hepatitis C virus infection. WHO. Published 2018. Accessed August 24, 2018. http://www.who.int/hepatitis/publications/hepatitis-c-guidelines-2018/en/

27. WHO. WHO | Health service delivery costs. WHO. Accessed June 5, 2020. http://www.who.int/choice/cost-effectiveness/inputs/health_service/en/

28. WHO. WHO | Global Price Reporting Mechanism for HIV, tuberculosis and malaria. WHO. Accessed March 19, 2020. https://www.who.int/hiv/amds/gprm/en/

29. Médecins Sans Frontières. Hepatitis C - Not even close. Médecins Sans Frontières Access Campaign. Accessed June 21, 2019. https://msfaccess.org/hepatitis-c-not-even-close

30. WHO. WHO | Progress report on access to hepatitis C treatment. WHO. Published 2018. Accessed September 17, 2019. http://www.who.int/hepatitis/publications/hep-c-access-report-2018/en/

31. Briggs AH, Goeree R, Blackhouse G, O’Brien BJ. Probabilistic analysis of cost-effectiveness models: choosing between treatment strategies for gastroesophageal reflux disease. *Med Decis Mak Int J Soc Med Decis Mak*. 2002;22(4):290-308. doi:10.1177/0272989X0202200408

32. Wilson EB. Probable Inference, the Law of Succession, and Statistical Inference. *J Am Stat Assoc*. 1927;22(158):209-212. doi:10.1080/01621459.1927.10502953

33. Efron B. Bootstrap Methods: Another Look at the Jackknife. *Ann Stat*. 1979;7(1):1-26. doi:10.1214/aos/1176344552

34. Landau HJ. *Moments in Mathematics*. American Mathematical Soc.; 1987.

35. Brown LD, Cai TT, DasGupta A. Interval Estimation for a Binomial Proportion. *Stat Sci*. 2001;16(2):101-133. doi:10.1214/ss/1009213286

36. Schnabel RB, Koonatz JE, Weiss BE. A modular system of algorithms for unconstrained minimization. *ACM Trans Math Softw*. 1986;11(4):419-440. doi:10.1145/6187.6192

37. R Core Team. *Non-Linear Minimization*.; 2019. Accessed July 4, 2019. https://stat.ethz.ch/R-manual/R-devel/library/stats/html/nlm.html

38. Kaplan DE, Chapko MK, Mehta R, et al. Healthcare Costs Related to Treatment of Hepatocellular Carcinoma Among Veterans With Cirrhosis in the United States. *Clin Gastroenterol Hepatol Off Clin Pract J Am Gastroenterol Assoc*. 2018;16(1):106-114.e5. doi:10.1016/j.cgh.2017.07.024

39. Schwarzinger M, Deuffic-Burban S, Mallet V, et al. 49 lifetime costs attributable to chronic hepatitis c from the french healthcare perspective (ANRS N°12188). *J Hepatol*. 2013;58:S21-S22. doi:10.1016/S0168-8278(13)60051-3

40. El Khoury AC, Klimack WK, Wallace C, Razavi H. Economic burden of hepatitis C-associated diseases in the United States. *J Viral Hepat*. 2012;19(3):153-160. doi:10.1111/j.1365-2893.2011.01563.x

41. McAdam-Marx C, McGarry LJ, Hane CA, Biskupiak J, Deniz B, Brixner DI. All-cause and incremental per patient per year cost associated with chronic hepatitis C virus and associated liver complications in the United States: a managed care perspective. *J Manag Care Pharm JMCP*. 2011;17(7):531-546. doi:10.18553/jmcp.2011.17.7.531
